# Bioactivity-Guided Fractionation, Characterization, and Mechanistic Insights of Anticancer Agents from *Simarouba glauca* DC. Leaves

**DOI:** 10.3390/molecules31030497

**Published:** 2026-01-31

**Authors:** Sushma Rudraswamy, Yashaswini Devi G. V., Sreeshyla H. Sheshanna, Nagabhushana Doggalli, SubbaRao V. Madhunapantula

**Affiliations:** 1 Department of Public Health Dentistry, JSS Dental College and Hospital, JSS Academy of Higher Education & Research, Mysore 570015, Karnataka, India; yashugv@gmail.com; 2 Department of Oral Pathology and Microbiology, JSS Dental College and Hospital, JSS Academy of Higher Education & Research, Mysore 570015, Karnataka, India; dr.sreeshylahs@jssuni.edu.in; 3 Department of Oral Medicine and Radiology, JSS Dental College and Hospital, JSS Academy of Higher Education & Research, Mysore 570015, Karnataka, India; dr.nagabhushand@jssuni.edu.in; 4 Department of Biochemistry, Center of Excellence in Molecular Biology and Regenerative Medicine, JSS Medical College, JSS Academy of Higher Education & Research, Mysore 570015, Karnataka, India

**Keywords:** *Simarouba glauca*, laxmitaru, anticancer activity, bioactivity-guided fractionation, oral squamous cell carcinoma (OSCC)

## Abstract

Although *Simarouba glauca* DC. has been recognized for its therapeutic properties, its anticancer effects against oral cancer have not been adequately investigated. The present study aimed to evaluate the activity of *S. glauca* leaf extracts against oral squamous cell carcinoma (OSCC). *S. glauca* leaves were extracted using solvents of increasing polarity, and the resulting fractions were evaluated for their phytochemical composition, antioxidant activity, and cytotoxic effects. Among all extracts, the *S. glauca* hexane extract (SGHE) exhibited the most potent anticancer activity against cell lines representing OSCC (CAL-27), cervical cancer (HeLa), and mouse mammary tumors (4T1). Bioactivity-guided fractionation identified D-erythro-Sphinganine as a major constituent present in hexane extract, possibly contributing to anticancer activity. But since the anticancer activity of crude hexane extract is superior compared to isolated D-erythro-Sphinganine, we predict a synergistic interaction among the multiple bioactive compounds present in the crude hexane extract. Hence, further studies were carried out with crude hexane extract. Mechanistic studies have shown that the anticancer activity of hexane extract is due to its ability to (a) alter cell cycle progression, (b) trigger apoptosis, and (c) inhibit cell migration in CAL-27 cells. Overall, these findings indicate that the hexane extract of *S. glauca* leaf possesses multi-target anticancer potential and warrants further mechanistic and in vivo investigations.

## 1. Introduction

Cancer is a major health crisis and is one among the leading causes of mortality worldwide. It was responsible for 10 million deaths in the year 2020, equating to nearly one in six deaths. The 2022 global cancer statistics predict that, by the year 2050, the number of new cases is likely to reach 35 million, which is a rise of 77% compared to the number of cases observed in the year 2022. In India, the most prevalent cancers among men are oral and lung cancers, while breast cancer is the most common among women [1,2]. Despite advances in early diagnosis and targeted therapy, cancer, being the deadliest disease with uncontrolled cell proliferation, continues to show poor prognoses and resistance to conventional chemotherapeutic agents in advanced stages. Moreover, current treatment options are frequently inadequate in combating the disease and are often associated with significant side effects, reduced quality of life, and high recurrence rates [3]. Thus, there is a growing emphasis on developing therapeutic compounds from plant sources that offer fewer side effects and greater efficacy in cancer treatment.

For thousands of years, plants have served as a source of medicine. Their diverse applications stem from the presence of compounds that possess therapeutic properties [4]. Although synthetic drugs are gaining popularity, plant-derived secondary metabolites serve as valuable templates for novel compounds. It is estimated that over 250,000 plant species exist on Earth, yet only about 10% have been researched [5]. Approximately one-third of the top-selling drugs worldwide are derived from natural compounds or their derivatives [6]. Prominent examples of plant-derived anticancer medications that are either in clinical use or undergoing trials include paclitaxel, vinca alkaloids, vincristine, vinblastine, resveratrol, curcumin, epigallocatechin gallate (EGCG), quercetin, rutin, betulinic acid, and artesunate [6,7]. In this context, the present study investigated the anticancer properties of *S. glauca*, also known as the paradise tree or laxmitaru. This tropical plant, belonging to the *Simaroubaceae* family, has been traditionally employed to treat ailments such as malaria, fever, dysentery, and cancer [8]. Numerous scientific studies support its pharmacological properties, which include antibacterial, antifungal, antioxidant, and anticancer effects [8,9]. The active compounds contributing to these effects are found in various parts of the plant: the leaves are high in terpenoids, flavonoids, and triterpenes; the bark contains quassinoids and alkaloids; the seeds are composed of fatty acids and fixed oils; and the root bark is recognized for its potent quassinoids that exhibit cytotoxic properties [10,11,12,13]. Quassinoids such as ailanthinone, glaucarubinone, and holacanthone have been abundantly reported in *S. glauca* and are recognized for their potent biological activities, including anticancer, antiprotozoal, antimalarial, and anti-amebic effects. In addition, β-carboline alkaloids, especially canthin-6-one derivatives, are widely distributed in the *Simaroubaceae* family and are reported to possess antiviral, cytotoxic, antiparasitic, antibacterial, and anti-inflammatory properties. Steroidal compounds have also been isolated from several genera of *Simaroubaceae*, emphasizing the chemical diversity and pharmacological potential of this family [14,15,16].

Various solvent extracts of *S. glauca* leaves, wood, and bark have demonstrated notable antioxidant activity in standard in vitro assays, including DPPH, ABTS, and FRAP. Methanolic and ethanolic extracts have been reported to consistently exhibit higher free radical scavenging and reducing capacity compared to other solvent fractions, which has been attributed primarily to the presence of terpenoids, phenolics, flavonoids, and fatty acid derivatives [17,18].

In addition, *S. glauca* extracts have demonstrated anticancer activity in several in vitro models. The methanolic extract of the leaf has been shown to exhibit significant cytotoxicity against the MCF-7 breast cancer cell line with an IC_50_ value of 16.12 µg/mL [19]. Similarly, the butanol fraction of *S. glauca* wood inhibited ~80% of murine embryonic stem cells, human prostate carcinoma (PC-3), and human embryonal carcinoma (NT-2) cells at 50 µg/mL. A bioactive compound isolated from the methanolic extract, laucarubol, was reported to suppress the growth of the renal carcinoma cell line RXF-393 [9]. Furthermore, activity-guided fractionation of chloroform-soluble extracts of *S. glauca* twigs yielded canthin-6-one alkaloid derivatives that exhibited cytotoxicity against multiple human cancer cell lines, including lung carcinoma [20]. Despite these promising observations, reported anticancer efficacy varies with extract type, concentration, and experimental model, and some in vivo studies indicate limited tumor regression, despite favorable safety profiles [21]. Although studies directly linking *S. glauca* to oxidative stress mechanisms are limited, Tricaprion, isolated from *S. glauca* leaves, has been reported to increase intracellular reactive oxygen species (ROS) levels, thereby inducing apoptosis and cancer cell death. Since histone deacetylase (HDAC) inhibition is known to promote ROS accumulation and oxidative-stress-mediated apoptosis; these findings suggest that oxidative-stress-related pathways may contribute to the anticancer activity of *S. glauca*-derived constituents [22].

Although *S. glauca* has been widely studied in different cancer models, research on oral cancer is scarce. Notably, a study reported that Glaucarubinone (GLU), a major quassinoid from *S. glauca*, enhances paclitaxel (PTX)-induced cytotoxicity in KB cells by activating apoptotic pathways involving p53, Bax, and Caspase-9, highlighting its potential role in reversing PTX resistance [23]. In addition, methanolic extracts of *S. glauca* leaves demonstrated selective cytotoxicity against SCC-9 oral cancer cells (IC_50_ = 312 µg/mL), with minimal or no activity in HCT116, MCF-7, and A549 cells, highlighting cell-line-specific anticancer effects [24].

In this context, the present study aimed to characterize the phytochemical profile, identify bioactive molecules, and evaluate the anticancer activities of *S. glauca* leaf extracts against cell lines representing OSCC, cervical cancer, and mouse mammary tumors. This work encompasses comprehensive analyses, including antioxidant assays, cytotoxicity evaluation, apoptosis and cell cycle studies, as well as LC–HRMS profiling to identify the potential bioactive compounds responsible for the observed effects. This integrated approach aims to elucidate the mechanistic basis of anticancer activity and highlight its relevance as a potential source of therapeutic lead compounds.

## 2. Results

### 2.1. Yield and Phytochemical Characterization of S. glauca Leaf Extracts

**Figure 1:**
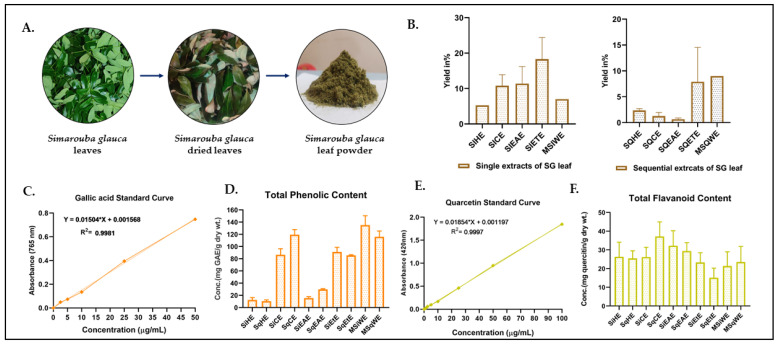
Extraction and phytochemical (TFC and TPC) estimation of *S. glauca* leaves. (**A**) Drying and pulverizing stages of *S. glauca* leaves. (**B**) The percentage yield for single extracts (SiHE, SiCE, SiEAE, SiETE, MSiWE. and sequential extracts (SqHE, SqCE, SqEAE, SqEtE, MSqWEz. (**C**) Gallic acid standard curve (0, 2.5, 5, 10, 25, and 50 µg/mL) for TPC. (**D**) TPC (in GAE/g dry wt. of the extract). (**E**) Quercetin standard curve (0, 2.5, 5, 10, 25, 50, and 100 µg/mL) for TFC. (**F**) TFC (in QE/g dry wt. of the extract) for the single and sequential extracts of *S. glauca* leaf. Each analysis was repeated three times, and the average values are plotted with standard deviations.

The dried leaves of *S. glauca* were extracted individually and successively with solvents of increasing polarity, including hexane (SiHE for single hexane extract and SqHE for sequential hexane extract), chloroform (SiCE for single chloroform extract and SqCE for sequential chloroform extract), ethyl acetate (SiEAE for single ethyl acetate extract and SqEAE sequential ethyl acetate extract), ethanol (SiEtE for single ethanol extract and SqEtE for sequential ethanol extract), and water (MSiWE for macerated single water extract and MSqWE for macerated sequential water extract). Among the various extracts obtained, SiCE and SiEtE had the highest yield of 13% and 14% with respect to the single extract. In the case of the sequential procedure, SqEtE and MSqWE had highest yield, i.e., 3.2% and 9.0%, respectively (Figure 1B).

Analysis of the extracts for various phytochemicals was performed as detailed in the Materials and Methods Section 4.2, and the results are expressed as a percentage of phytochemicals (quercetin equivalents, QE, in the case of flavonoids and Gallic acid equivalents, GAE, in the case of TPC) per g of extract. The findings reveal that SqCE had the highest flavonoid content, i.e., 4.2% QE. The order of TFC was found to be SqCE > SiEAE > SqEAE > MSqWE > SiCE > SiEtE > MSiWE > SiHE > SqHE > SqEtE (Figure 1E). The TPC varied from 0.1% to 14.3%, respectively, in SiHE to MSqWE (Figure 1D). Very low TPC was observed in single and sequential extracts of hexane and chloroform. The TPC was greater in the ethyl acetate, ethanol, and water extracts in the order of MSiWE > SqEAE > MSqWE > SiEtE > SiEAE > SqEtE.

After the determination of TPC and TFC, the extracts were subjected to analysis by RP-HPLC to measure the relative percentages of known phytochemicals. The identification of compounds was performed by comparing the recorded retention time (RT) for the extracts with standards of benzoic acids and cinnamic acids. The obtained peaks from all the extracts revealed the presence of Gallic acid, Protocatechuic acid, p-Hydroxy benzoic acid, Gentisic acid, Vanillic acid, Syringic acid, Veratric acid, Eudesmic acid, Benzoic acid, p-coumaric acid, Caffeic acid, Ferulic acid, Sinapic acid, Trans Cinnamic acid, 3,4- Dimethoxy cinnamic acid, and Chlorogenic acid. The single and sequential extracts of ethyl acetate, ethanol, and water had higher levels of these phenolic compounds compared to other hexane and chloroform extracts (Figure S2). The obtained RP-HPLC findings even correlated with the quantitative TPC results.

### 2.2. Antioxidant Potential of S. glauca Leaf Extracts

**Figure 2:**
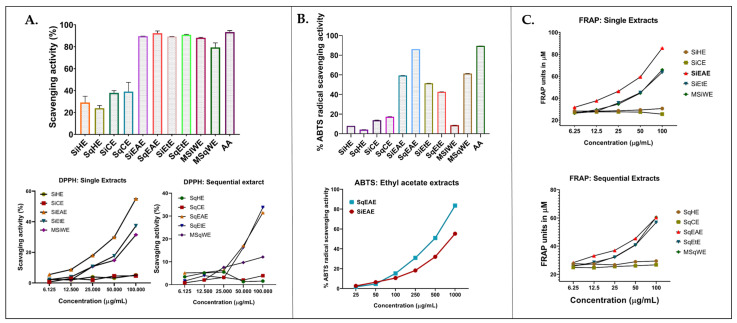
Antioxidant activity of the extracts generated from *S. glauca* leaves. (**A**) DPPH assay for single and sequential extracts at 1.0 mg/mL and at different doses ranging from 6.125 to 100 µg/mL. (**B**) ABTS assay for single and sequential extracts at 1.0 mg/mL. Second panel in (**B**) shows the dose response study data of ethyl acetate extracts at different concentrations (25–1000 µg/mL). (**C**) FRAP assay for single and sequential extracts at 1.0 mg/mL and at different concentrations (6.125–100 µg/mL). All data represent the mean ± SD of three independent experiments.

The antioxidant activity of *S. glauca* leaf extracts was evaluated using multiple in vitro assays [25], which include radical scavenging activity by testing against the DPPH and ABTS radicals and reduction power by measuring the Ferric ion Reduction Antioxidant Power (FRAP) (Figure 2). As each assay is based on distinct reaction mechanisms and radical systems, direct comparisons of their quantitative outcomes are likely to differ [26]. The initial screening of the radical scavenging ability was performed using 1.0 mg/mL of extracts. Extracts of ethyl acetate (SiEAE and SqEAE), ethanol extracts (SiEtE and SqEtE), and water (MSiWE and MSqWE) showed higher radical scavenging activity compared to the extracts generated using hexane (SiHE and SqHE) and chloroform (SiCE and SqCE).

The DPPH radical scavenging assay showed that all *S. glauca* leaf extracts exhibited a dose-dependent increase in radical scavenging potential across the tested concentrations (6.125–100 µg/mL), as represented in Figure 2A. Among the extracts, the single ethyl acetate extract (SiEAE) showed the highest activity with an IC_50_ value of 89.5 µg/mL, followed by the sequential ethanol (SqEtE, 153.6 µg/mL) and single ethanol extract (SiEtE, 154.6 µg/mL). The overall scavenging potential decreased in the following order: chloroform < hexane < water < ethanol < ethyl acetate for both single and sequential extractions. The ABTS assay also revealed significant differences among the extracts (Figure 2B), wherein both single and sequential ethyl acetate extracts displayed markedly higher scavenging activity than the other extracts. Further dose-dependent analysis (25–1000 µg/mL) confirmed enhanced activity, particularly for the sequential ethyl acetate extract (SqEAE), which exhibited an IC_50_ value of 424.4 µg/mL. Similarly, in the FRAP assay, both single and sequential extracts showed concentration-dependent increases in reducing power (6.125–100 µg/mL). The ethyl acetate extracts (SiEAE and SqEAE) demonstrated the highest reducing potential with IC_50_ values of 22.93 and 57.35 µg/mL, respectively, following the same activity trend observed in the DPPH assay (Figure 2C).

### 2.3. Cytotoxicity Assessment of the Extracts Generated from S. glauca Leaves

The cytotoxic potential of *S. glauca* leaf extracts was evaluated against three cancer cell lines—oral squamous cell carcinoma (CAL-27), cervical cancer (HeLa), and breast cancer (4T1)—using an MTT assay at 24 h and 48 h. The cell lines were selected as representative and well-established models of aggressive epithelial cancers commonly used for cytotoxic and anticancer screening, with 4T1 serving as a cell line referring to highly metastatic breast cancer. The obtained results reveal notable differences in extract activity among the cell lines, as summarized in Table 1.

In the case of oral squamous cell carcinoma (CAL-27), the hexane extract (SiHE) exhibited the highest cytotoxicity, followed by the sequential ethanol, chloroform, and ethyl acetate extracts, with IC_50_ values of 142, 108, 131, and 230.3 μg/mL, respectively. Among the single extracts, the ethyl acetate extract (SiEAE) demonstrated comparatively better activity with an IC_50_ value of 189 μg/mL.

When tested against cervical cancer (HeLa) cells, the single water extract (SiWE) was found to be the most potent, with an IC_50_ value of 265 μg/mL, followed by the hexane extract (SiHE) at 299 μg/mL and the sequential ethyl acetate extract (SqEAE) at 742 μg/mL. The strong cytotoxic response of the water extract in HeLa cells suggests the presence of polar bioactive molecules capable of modulating the pathways involved in the regulation of cervical carcinoma cell viability. The results of the cytotoxicity assessment against the mouse breast cancer cell line 4T1 show that the single ethanol extract (SiEtE) exhibited the greatest potency, with an IC_50_ of 132.7 μg/mL, followed by the hexane extract (SiHE) and sequential ethyl acetate extract (SqEAE) with IC_50_ values of 256 μg/mL, 230 μg/mL, and 542 μg/mL, respectively.

The cytotoxic effect of the extracts was also evaluated in BEAS-2B cells to assess their selectivity toward cancer cells (Figure S3). At lower concentrations (62.5–125 µg/mL), the extracts exhibited negative cytotoxic values, indicating an absence of toxicity and a possible proliferative response in normal cells. However, with increasing concentrations (250–1000 µg/mL), a gradual reduction in cell viability was observed for all extracts, confirming a concentration-dependent effect. These findings, presented in the Supplementary Data, indicate that *S. glauca* leaf extracts exhibit minimal cytotoxicity toward the normal cell line, as the obtained IC_50_ values were >500 µg/mL [27].

### 2.4. Characterization of the Hexane Extract Exhibiting Potent Cytotoxicity by LC-HRMS

**Figure 3:**
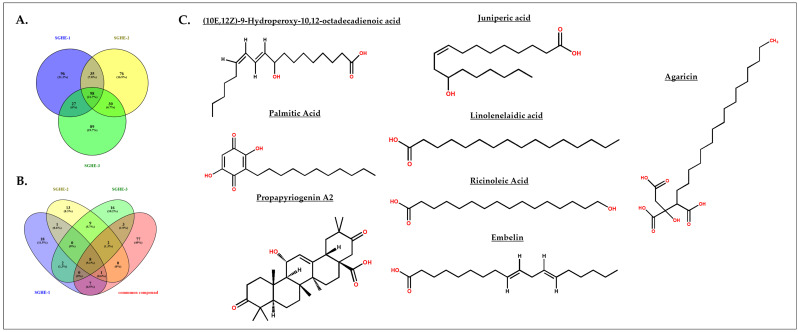
Chemical structures of identified compounds in hexane extract of *S. glauca* leaf using LC-HRMS: (**A**) Common compounds present in the three replicate batches of hexane extract of *S. glauca* leaf. (**B**) Primary metabolites present among the triplicates and common compounds after the removal of synthetic compounds. (**C**) Structure of the obtained primary metabolites in hexane extract of *S. glauca* leaves, with functional groups highlighted in red.

LC–HRMS analysis was carried out to characterize the phytochemical constituents present in the hexane extract of *S. glauca* leaves. The chromatographic profile exhibited several well-resolved peaks corresponding to compounds of different retention times and molecular weights, confirming the complex chemical nature of the extract. To ensure analytical reproducibility, three independent replicates (SGHE-1, SGHE-2, and SGHE-3) were analyzed, and the common compounds present in these three replicate batches were identified (Figure 3A). The selection of predominant constituents was based on the common peaks shared among the replicates, followed by the elimination of synthetic compounds, and retention of natural metabolites with higher abundance. The comparative Venn diagrams (Figure 3B) highlight the overlapping metabolites among the triplicates, indicating the consistency and reproducibility of compound detection. Eight major natural compounds are identified as predominant constituents of the extract listed with RT, molecular weight, chemical formula, and classification in Table S1 and chemical structure in Figure 3C. These compounds belong to diverse phytochemical classes, including oxylipins, quinones, fatty acids, and triterpenoids. Among them, (10E,12Z)-9-hydroperoxy-10,12-octadecadienoic acid (C_18_H_32_O_4_; *m*/*z* 313.23; RT 8.82 min) was detected as a major oxylipin-type hydroperoxy fatty acid, which is often associated with antioxidant and anticancer properties. Embelin (C_17_H_26_O_4_; *m*/*z* 295.18; RT 8.67 min), a Quinone derivative, was also found in significant abundance and is well documented for its potent anticancer and antioxidant activities.

The extract additionally contained Ricinoleic acid (C_18_H_34_O_3_; *m*/*z* 299.25; RT 10.84 min), a hydroxylated fatty acid known for its anti-inflammatory potential, along with Linolenelaidic acid (C_18_H_30_O_2_; *m*/*z* 279.22; RT 10.84 min), an unsaturated fatty acid exhibiting antioxidant and membrane-modulating properties. The presence of Juniperic acid (C_16_H_32_O_3_; *m*/*z* 273.23; RT 10.09 min) and palmitic acid (C_16_H_32_O_2_; *m*/*z* 257.24; RT 12.17 min) reflects the contribution of long-chain and saturated fatty acids to the extract composition, indicating the lipophilic nature of SGHE.

Two triterpenoid derivatives, Propapyriogenin A2 (C_30_H_44_O_5_; *m*/*z* 485.32; RT 10.01 min) and Agaricin (C_22_H_40_O_7_; *m*/*z* 417.28; RT 9.63 min), were also identified as bioactive constituents. These compounds are reported to possess cytotoxic and antiproliferative properties, suggesting their possible contribution to the observed biological effects of the extract. The presence of both triterpenoid and fatty acid derivatives indicates that the hexane extract predominantly contains non-polar bioactive molecules, which may synergistically contribute to its antioxidant and anticancer potential.

### 2.5. Bioactivity Guided Fractionation and Enrichment with Chemical Characterization of Active Fraction

In order to identify the key constituents responsible for the cytotoxicity of hexane extract, the sample was fractionated as detailed in the Materials and Methods Section 4.6 and the concentrated fractions were tested for bioactivity using the CAL-27 cell line by an MTT assay, as explained earlier. The fraction 44–55 showed greater activity, as represented in Figure S5. The active fraction was chemically characterized using LC-MS, ^1^H, and ^13^C NMR.

**Figure 4:**
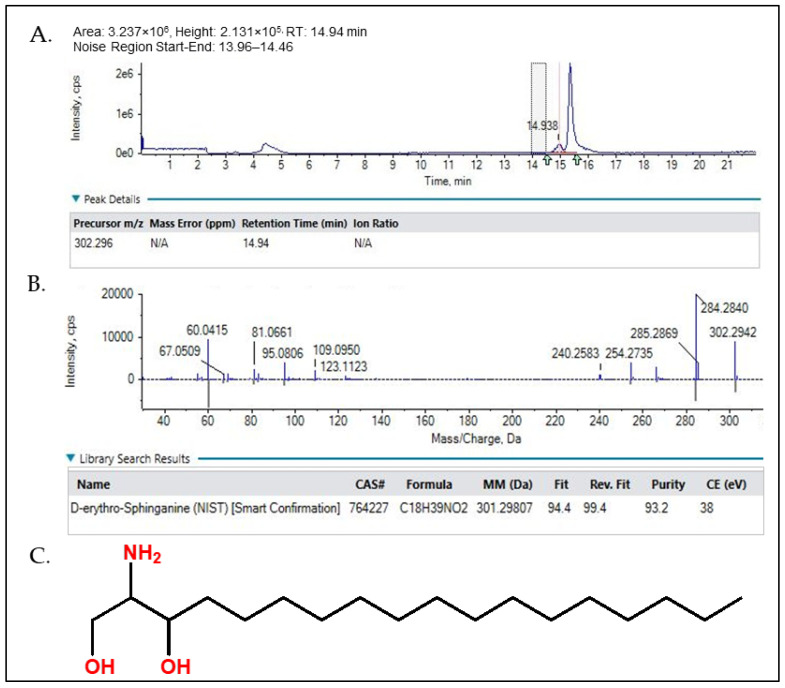
Chemical characterization of the active fraction (44–55) of the hexane extract of S. glauca leaves. (**A**) Extracted ion chromatogram (XIC) showed a precursor ion at *m*/*z* 302.296 [M + H]^+^ with a retention time of 14.94 min. The arrow indicates the detected analyte peak (RT 14.94 min), the dotted box represents the noise/baseline region used for signal-to-noise calculation, and colored markers denote software-assigned peak integration features. (**B**) MS/MS spectrum acquired at a collision energy of 25 eV exhibited characteristic fragment ions at *m*/*z* 284, 285, 254, and 240. (**C**) Chemical structure of D-erythro-Sphniganine, red highlight represent the functional group within the structure.

**Figure 5:**
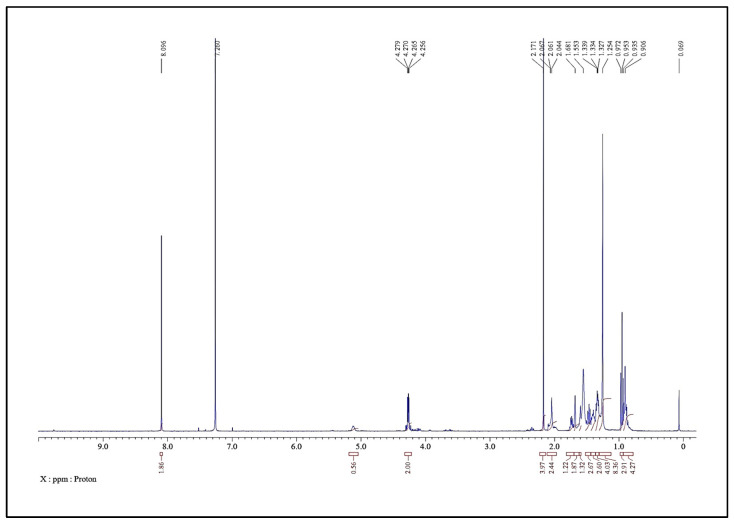
Chemical characterization of the active fraction (44–55) of the hexane extract of *S. glauca* leaves using ^1^H-NMR.

**Figure 6:**
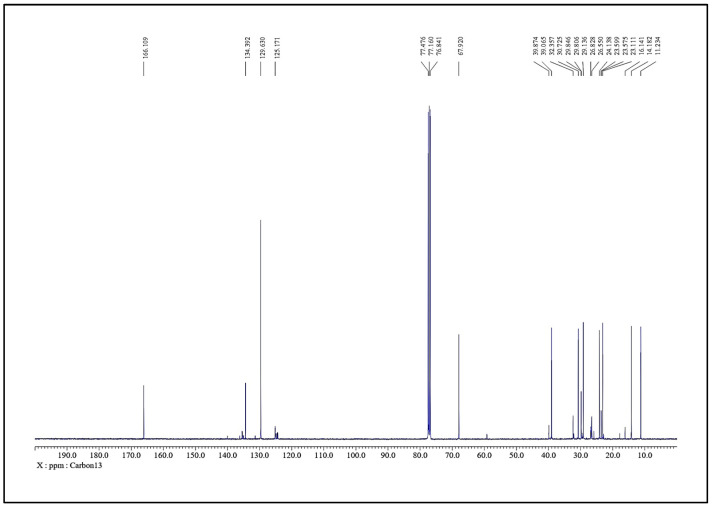
Chemical characterization of the active fraction (44–55) of the hexane extract of *S. glauca* leaves using ^13^C-NMR.

The obtained LC-HRMS results, as shown in Figure 4, match with the standard D-erythro Sphinganine with 93–94% purity. Thus, the final fraction 44–55 was most effectively composed of 93–94% D-erythro Sphinganine and 6–7% unidentified minor constituents. As a natural product, the chemical composition of *S. glauca* leaf extracts may vary depending on geographical origin, season, and harvesting period, which could influence the relative abundance of D-erythro-sphinganine and consequently affect the observed IC_50_ values [28,29,30]. The obtained ^1^H and ^13^C NMR spectra were also correlated with the LC-MS results. The NMR spectra of the material confirm D-erythro-Sphinganine or D-erythro-dihydrosphingosine as the major component (~93%) with minor constituents (~6%). In the case of the analysis using ^1^H-NMR (400 MHz, CDCl_3_) (Figure 5), a singlet at δ 8.10 ppm (2 H) corresponds to amide/imine NH, a multiplet at δ 5.04–5.19 ppm (1 H) represents the vinylic proton, and doublets at δ 4.27 ppm (2 H) are assignable to CH_2_ adjacent to hydroxyl or amine. Aliphatic signals include δ 2.17–2.04 ppm (6 H, CH_2_ near carbonyl or double bond), δ 1.73–1.60 ppm (4 H, methine/CH_2_ adjacent to polar groups), δ 1.50–1.24 ppm (18 H, long-chain CH_2_), and terminal methyl at δ 0.95–0.86 ppm (7 H). The ^13^C-NMR (100 MHz, CDCl_3_) (Figure 6) shows the carbonyl at δ 166.1 ppm, aromatic/alkenic carbons at δ 134.4–125.2 ppm, oxygenated carbons at δ 77.5–67.9 ppm, methines near electronegative atoms at δ 39.9–39.1 ppm, long-chain methylenes at δ 32.4 ppm, and terminal methyl carbons at δ 16.1 ppm. Together, the ^1^H and ^13^C NMR data confirm a composition dominated by D-erythro-Sphinganine with minor co-constituents, consistent with the stated purity and suitable for pharmaceutical formulation.

Bioactivity was tested for both effective fraction and the standard D-erythro Sphinganine (D3314, Sigma Aldrich, St. Louis, MO, USA) by Sulforhodamine B. The results show that the active fraction and standard compound exhibited approximately 50% cell death at a concentration of 1 mg/mL, indicating that the observed activity of the fraction is predominantly attributable to its major constituent, D-erythro-sphinganine (Figure S6). These results suggest that the fraction does not exhibit any significant synergistic or additive effect.

### 2.6. Treatment of Cancer Cells with S. glauca Leaf Hexane Extract Induced Death—Analysis by Live–Dead Staining Assay

**Figure 7:**
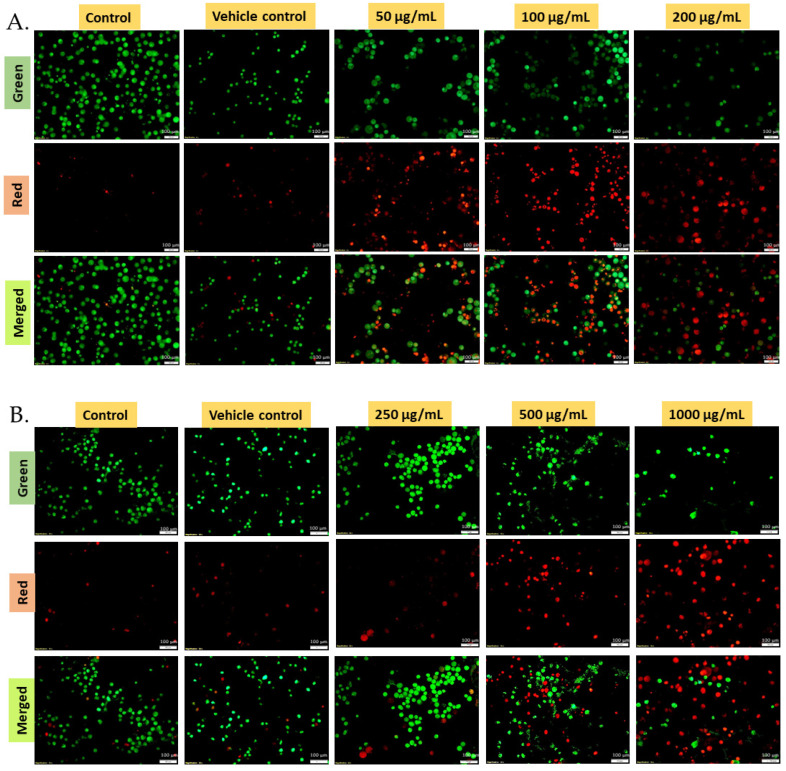

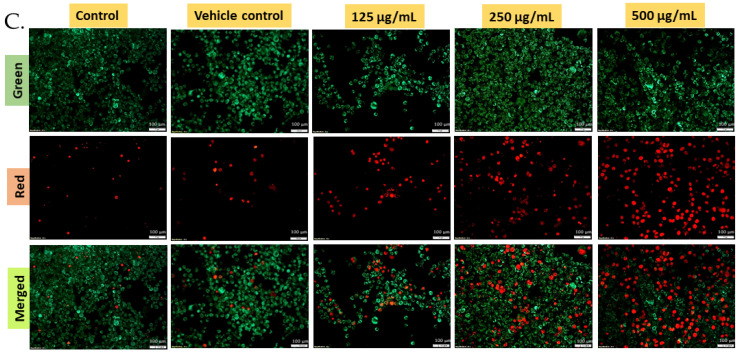
Live–dead staining for the hexane extracts of *S. glauca* leaf: acridine orange and ethidium bromide staining images using a fluorescent microscope at a magnification of 20×. Images represent the photomicrographs of cells exposed to *S. glauca* leaf hexane extract, which triggered apoptosis in (**A**) Cal-27, (**B**) HeLa, and (**C**) 4T1.

The fluorescence images of CAL-27, HeLa, and 4T1 cell lines treated with different concentrations of hexane extract are represented in Figure 7A–C, respectively. The assay is based on the ability of (a) acridine orange (AO) to stain both live and dead cells green and (b) ethidium bromide (EtBr) to penetrate only the compromised membranes of necrotic or apoptotic cells, staining them red. The merged images therefore distinguish viable cells (green fluorescence) from apoptotic or necrotic cells (red fluorescence) [31]. A concentration-dependent increase in the number of red-stained cells was observed in all three cell lines following treatment with the extract, indicating a progressive loss of membrane integrity and cell death. The predominance of red-stained cells at higher concentrations suggests late apoptosis or necrosis as the dominant mode of cell death [32]. In contrast, untreated control and vehicle control (1.0% DMSO) cells exhibited predominantly green fluorescence, confirming cell viability and the absence of cytotoxic effects.

### 2.7. Treatment of Cancer Cells with S. glauca Hexane Extract Arrested the Progression of the Cell Cycle by Promoting the Accumulation of Cells in the G_2_/M Phase

**Figure 8:**
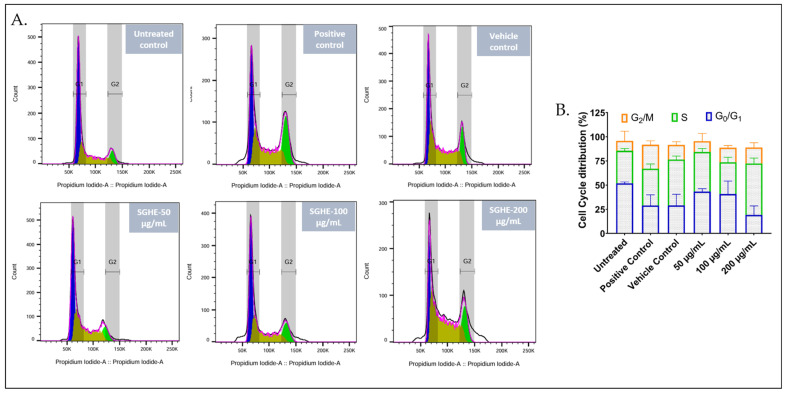
Cell cycle analysis: The CAL-27 cells were treated with hexane extract at 50, 100, and 200 μg/mL, vehicle (1% DMSO), and Camptothecin (25 nM) for 24 h. (**A**) The treated cells were analyzed by flow cytometry to determine the percentage cells in each phase of cell cycle. Colors indicate cell-cycle phases: G_1_ (blue), S (brown), and G_2_/M (green). Grey shaded areas represent gating regions (**B**) Quantification of a cell population (in %) in different phases of the cell cycle is shown as bar diagrams. All data represent the mean ± SD.

The effect of the hexane extract of *S. glauca* leaf was examined by performing flow cytometry analysis of propidium iodide-stained CAL-27 cells after 24 h of treatment at different concentrations (50, 100, and 200 μg/mL), as represented in Figure 8. The untreated cells with 51.8% cells in the G_1_ phase, 33.7% in the S phase, and 10.3% in the G_2_/M phase reflected normal proliferative profile [33]. The cells treated with *S. glauca* hexane extract displayed a variation in the phases of distribution among the treated concentrations. The vehicle control treated cells showed a slight elevation in the S phase population, which could be due to solvent-induced stress. The cells exposed to 50 µg/mL extract exhibited G_1_/S phase transition interference [34]. At 100 µg/mL, a decrease in the G_0_/G_1_ phase and a simultaneous increase in the G_2_/M phase, with a slight decrease in the S phase population, was observed, which indicated G_2_/M cell cycle arrest [35]. At the highest concentration, i.e., 200 µg/mL, elevation in the S phase population and an increase in the G_2_/M phase, along with a sharp decrease in the G_0_/G_1_ phase, indicate a shift in the cells from the resting to the active proliferation phase [36]. In the case of the positive control, Camptothecin (25 nM), a noticeable decrease in the G_0_/G_1_ phase with a slight increase in the S phase and elevated G_2_/M phase was observed, which suggests a G_2_/M phase arrest [37].

### 2.8. S. glauca Hexane Extract Induced Apoptosis in CAL-27 Cells

**Figure 9:**
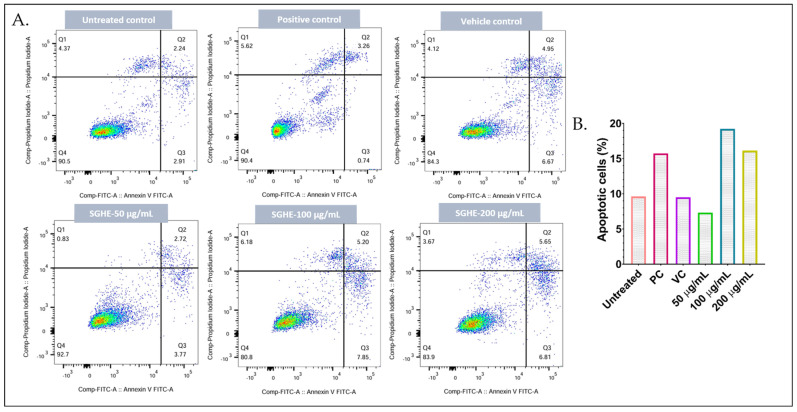
Apoptosis analysis: The CAL-27 cells were treated with the different concentrations of hexane extract at 50, 100 and 200 μg/mL, vehicle, and Camptothecin (25 nM) for 24 h. The treated cells were stained by incubating with annexin V–FITC and PI the obtained. (**A**) Scatterplots, represent Quadrants indicating viable, early apoptotic, late apoptotic, and necrotic cell populations. Color intensity represents event density (blue = low, red = high). (**B**) Graphical representation of the percentage of apoptotic cells.

The induction of apoptotic cell death is one of the key mechanisms reported for many anticancer agents. Therefore, the apoptosis-inducing effect of *S. glauca* hexane extract was determined by treating CAL-27 cells with increasing concentrations of the sample followed by treating the control. After the treatment period, both control and extract-treated cells were stained with annexin V-FITC and PI and analyzed by flow cytometry (Figure 9). The percentage of apoptotic cell population (early and late apoptotic cells) increased in the treatment groups compared to the control cells. The untreated and vehicle control (1.0% DMSO) groups exhibited predominantly viable cells. The CAL-27 cells treated with 50 µg/mL extract showed a pattern similar to untreated cells with 3.77% early apoptosis cells, but the higher concentrations of the extract, viz., 100 and 200 µg/mL, displayed 7.85 and 6.81% of early apoptotic cells and 5.2 and 5.65% of late apoptotic cells. The positive control showed 6.67% of early apoptotic and 4.95% of late apoptotic cells. Overall, treatment with the hexane extract promoted a moderate but significant increase in apoptotic cell population.

### 2.9. CAL-27 Cells Exposed to S. glauca Hexane Extract Exhibited Retarded Migration

**Figure 10:**
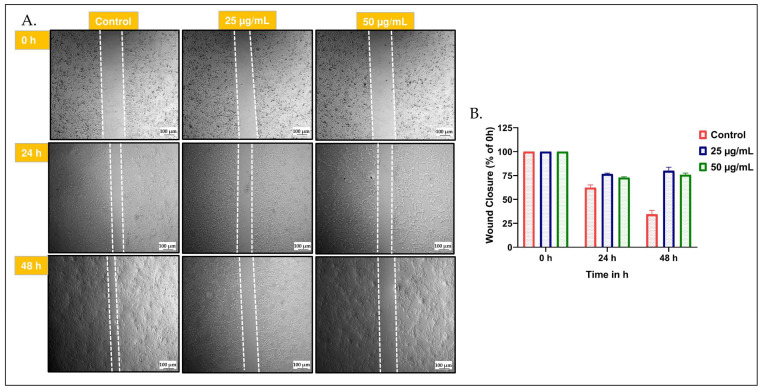
Photomicrographs depicting the migration assay results: The CAL-27 cells were treated with different concentrations of *S. glauca* hexane extract at 25 and 50 μg/mL. (**A**) The images were captured at 0, 24, and 48 h. The region between the dashed line represent the area of migration. (**B**) Quantification was performed using Image J software (1.54).

The migration of cells from primary tumors to secondary sites is one of the typical characteristics of cancer cells. Pharmacological agents inhibiting/retarding this migration helps in reducing the metastatic spread of tumor cells. In order to test whether the *S. glauca* hexane extract has the potential of inhibiting/retarding the CAL-27 cells, a wound healing assay was carried out, as detailed in the Materials and Methods section (Figure 10). At 0 h, all tested groups displayed a uniform scratch width, confirming consistent wound initiation. In the control group, progressive closure of the wound area was observed at 24 h and nearly complete closure at 48 h, indicating normal migratory behavior. However, treatment with *S. glauca* leaf hexane extract significantly reduced wound closure in a concentration- and time-dependent manner. Cells treated with non-toxic 25 μg/mL extract showed partial inhibition of migration. In contrast, those treated with 50 μg/mL exhibited a substantial reduction in wound closure, with large unhealed gaps persisting even after 48 h. Quantitative analysis demonstrated that the wound closure percentage decreased from approximately 95% in the control to 62% and 35% at 25 μg/mL and 50 μg/mL, of hexane extract, respectively, after 48 h. These findings suggest that *S. glauca* leaf hexane extract not only causes cell death, but also effectively suppresses the migratory potential of CAL-27 cells.

## 3. Discussion

Our findings on the phytochemical profiles reveal a higher flavonoid content in the chloroform extract of *S. glauca* leaves, which is consistent with earlier reports that recorded 4.195 ± 0.50 g% QE [38]. The solvent-dependent distribution of flavonoids suggests the presence of both glycosidic and aglycone forms, including flavanones, flavones, and isoflavones [39], emphasizing the critical role of solvent polarity in efficient extraction. Our experiments show that the selection of an appropriate extraction solvent depends on the type and polarity of the constituents in the plant material. Similarly, our RP-HPLC and TPC results correlate with previous reports demonstrating the influence of solvent polarity on phenolic compound extraction. Polar solvents such as ethyl acetate and ethanol have been shown to yield higher levels of phenolic compounds compared to hexane, chloroform, and water extracts [26,40]. José et al. 2020 reported the presence of 2,3,4-trihydroxybenzoic acid (THBA; Gallic acid), 3,4-dihydroxybenzoic acid (DHBA; Protocatechuic acid), and 3,4-dimethoxycinnamic acid (DMCA; Caffeic acid dimethyl ether) in ethyl acetate and ethanol extracts, contributing to their anticancer activity against the lung, cervical, breast, colon, and rectal cancer cell lines [41]. Collectively, the presence of these diverse secondary metabolites supports the strong antioxidant and antiproliferative potential of *S. glauca* leaf extracts [42].

The antioxidant results from DPPH, ABTS, and FRAP demonstrated greater scavenging potential in the order of Ethyl acetate > Ethanol > Water > Chloroform > Hexane. The results are in agreement with previous reports, which demonstrated potent antioxidant activity for the ethanol extract of *S. glauca* leaves, with an IC_50_ value of 108.8 µg/mL among organic and aqueous extracts. Similarly, another study reported DPPH radical scavenging activity in the order of acetone > methanol > chloroform > petroleum ether > water, indicating that mid-polar solvents possess greater antioxidant potential followed by more polar solvent extracts and substantially very low activity in the non-polar solvent extracts [19,38]. In support, several studies have reported notable antioxidant activity of *S. glauca* leaf extracts prepared using solvents of varying polarity. Aqueous, ethanol, and ethyl acetate extracts demonstrated antioxidant potential with IC_50_ values of 180.7 µg/mL, 209.7 µg/mL, and 678.2 µg/mL, respectively [18]. In another study, ethyl acetate and petroleum ether extracts of *S. glauca* leaves exhibited dose-dependent antioxidant activity, with IC_50_ values of 1000 µg/mL and 2000 µg/mL, respectively [43]. Similarly, Rudraswamy et al. reported strong antioxidant activity for the ethanolic leaf extract, with an IC_50_ value of 108.8 µg/mL, while the aqueous extract showed an IC_50_ value of 310 µg/mL [44].

Overall, a consistent trend is evident across these previous reports, as well as in the present study, wherein extracts obtained using relatively polar solvents exhibit superior antioxidant activity compared to those derived from non-polar or moderately polar solvents. This enhanced activity is likely attributable to the higher extraction efficiency of phenolic and flavonoid compounds in polar solvents, which are well recognized for their free radical scavenging properties.

The cytotoxic evaluation of *S. glauca* leaf extracts revealed distinct activity patterns among different solvent fractions and cancer cell types [45]. The non-polar hexane extracts (SiHE and SqHE) showed consistent and potent cytotoxicity across all tested cell lines, suggesting that bioactive constituents with anticancer potential are largely concentrated in the non-polar fraction. This observation agrees with previous reports highlighting that non-polar triterpenoids, quassinoids, and limonoids from *S. glauca* are major contributors to cytotoxic activity through mechanisms involving apoptosis and cell growth inhibition [9,46,47]. Overall, the pattern of IC_50_ values across cell lines indicates that *S. glauca* leaf contains multiple classes of bioactive molecules with distinct polarities and selectivities. The consistent efficacy of the hexane fractions reinforces the importance of non-polar compounds, while the activity of ethanol and water extracts reflects complementary mechanisms. Variations in cytotoxic response may be due to batch-to-batch differences in extraction efficiency and phytochemical composition, as supported by LC–MS analysis. These findings collectively support the broad-spectrum cytotoxic potential of *S. glauca* leaf extracts and provide a rationale for further mechanistic studies focusing on apoptosis and cell cycle modulation.

The combined cell cycle and apoptosis analyses provide mechanistic insights into the cytotoxic effects of the *S. glauca* hexane extract. The extract induced a marked alteration in cell cycle progression, characterized by a G_2_/M arrest at intermediate concentrations and an S-phase accumulation at higher doses. Such phase disruptions are indicative of DNA damage or inhibition of the cyclin–CDK complex, which consequently halts the cell division and triggers apoptotic signaling [48,49]. The concentration-dependent increase in early and late apoptotic populations further supports apoptosis as a primary mode of cell death rather than necrosis. These findings are in agreement with earlier reports where triterpenoid- and fatty-acid-rich plant extracts induced G_2_/M arrest and apoptosis through ROS generation and mitochondrial dysfunction [50,51,52].

The LC–HRMS profiling of SGHE supports the observed biological activities by revealing the presence of several non-polar bioactive compounds with potential to modulate key molecular pathways. Among them, Propapyriogenin A2 and Agaricin are the triterpenoids present. Propapyriogenin A2 has been reported to exhibit antihepatotoxic, anti-hyperglycemic, and anti-adipogenic properties, especially in *Lagerstroemia speciosa* (Banaba) leaf extracts, although its anticancer or antiproliferative potential has not yet been documented [53]. In contrast, Agaricin (or agaric acid) has been shown to interact with adenine nucleotide translocase (ANT), leading to mitochondrial dysfunction and oxidative stress. This interaction results in mitochondrial membrane damage and the generation of reactive oxygen species, ultimately promoting apoptosis [54,55].

Embelin, a naturally occurring benzoquinone, has been extensively reported for its multifunctional pharmacological activities, particularly in cancer prevention and therapy. Previous studies have demonstrated that Embelin inhibits TNF-α converting enzyme (TACE), thereby suppressing TNF-α–mediated signaling and reducing cancer cell invasion and metastasis [55]. Molecular dynamics and experimental studies confirm embelin’s strong affinity for TACE, correlating with its ability to modulate inflammatory and apoptotic pathways. These findings suggest that the presence of Embelin in SGHE may contribute to its observed antiproliferative and pro-apoptotic effects [56,57].

Additionally, the detection of 10-hydroxy-2-decenoic acid (10-HDA) in the LC–HRMS profile highlights another potential contributor to the anticancer activity of SGHE. 10-HDA, a bioactive fatty acid primarily found in royal jelly, has been shown to induce reactive oxygen species (ROS)-mediated apoptosis in various cancer cell lines. It regulates key signaling pathways, including MAPK, STAT3, NF-κB, and TGF-β1, leading to mitochondrial dysfunction and activation of apoptotic cascades. Therefore, the presence of 10-HDA in SGHE may play a significant role in promoting oxidative-stress-induced apoptosis and inhibiting cancer cell proliferation [58,59].

The identification of palmitic acid in SGHE is also noteworthy due to its established role in tumor biology. Palmitic acid, a common saturated fatty acid, has been implicated in modulating tumor progression through its involvement in membrane remodeling, lipid signaling, and protein palmitoylation. Recent studies suggest that palmitic acid can influence oncogenic signaling, inflammation, and tumor–nerve interactions [60], thereby promoting cancer cell survival and metastasis. Hence, its detection in SGHE may indicate a potential modulatory role in the lipid-mediated signaling pathways associated with tumor progression [61,62]. Finally, Ricinoleic acid, a hydroxylated fatty acid primarily derived from *Ricinus communis* L., was also detected. Ricinoleic acid, a hydroxylated fatty acid predominantly found in *Ricinus communis* L., has been reported to exhibit diverse pharmacological properties, including anticancer, anti-inflammatory, and pro-apoptotic effects [63]. Studies have shown that *Ricinus communis* fruit extract and Ricinoleic acid derivatives can inhibit cancer cell migration and invasion while inducing apoptosis and suppressing tumor progression. Moreover, Ricinoleic acid has demonstrated both pro- and anti-inflammatory actions, suggesting its ability to modulate multiple cellular pathways relevant to cancer therapy [64,65].

Together, these findings indicate that the cytotoxic potential of hexane extract likely arises from a synergistic interaction among multiple non-polar phytoconstituents, particularly triterpenoids, quinones, and fatty acid derivatives. Their collective action disrupts cell cycle progression, compromises membrane integrity, and promotes programmed cell death. The alignment between LC–HRMS-identified metabolites and the observed cellular responses strongly supports the role of non-polar bioactive as the principal anticancer agents in *S. glauca* leaf extract. Further mechanistic studies focusing on mitochondrial signaling, ROS modulation, and caspase activation would be valuable to delineate the precise molecular pathways underlying these effects.

## 4. Materials and Methods

### 4.1. Plant Material Collection

The leaves of *S. glauca* were collected in March 2024 from Suttur, Mysore, with GPS coordinates of 12.148° N and 76.7916° S. The plant was identified and authenticated by the Department of Studies in Botany, University of Mysore, Karnataka, India. The collected leaves were washed thoroughly, shadow-dried initially, and were brought to JSS College of Pharmacy, Mysore, where the leaves were dried in a hot-air oven at 45 °C and pulverized into powder using an electric grinder (camel, 1200 watts). The powder was sieved (no. 12, particle size 1.68 mm) before storing for future analysis.

#### Preparation and Extraction of *S. glauca* Leaves

The sequential extraction of *S. glauca* leaves was conducted using a Soxhlet apparatus (250 mL, Soxhlet extractor and 500 mL round-bottom flask) with solvents of increasing polarity, viz., hexane, chloroform, ethyl acetate, ethanol, and double-distilled water (Analytical grade solvents, purity > 99%, SRL, Mumbai) Experimentally, 25 g of ground *S. glauca* leaf powder was placed in a thimble and positioned in the extraction chamber. The round-bottom flask was filled with 150 mL of the chosen solvent (except water), and the extraction temperature was adjusted with a heating mantle to the boiling point of each solvent: 70 °C for hexane, 65 °C for chloroform, 70 °C for ethyl acetate, and 80 °C for ethanol. The resulting plant extracts were then collected in the round-bottom flask. The residue left after each solvent extract was further processed in a similar fashion, but with a subsequent solvent.

For single extraction, fresh *S. glauca* dried leaves were used for each solvent, and the extraction was carried out similarly to the one described in sequential method. The collected extracts were concentrated using a rotary evaporator.

The extraction yield was determined using the following formula:

Extraction yield (%) = (A/B) × 100(1)

where “A” is the weight of the dried extract and “B” is the dry weight of the *S. glauca* leaves.

### 4.2. Analysis of Phytochemicals

#### 4.2.1. Determination of Total Phenolic Content (TPC)

The Folin–Ciocalteu method was employed to quantify TPC in *S. glauca* leaf extracts [66]. In brief, increasing concentrations of Gallic acid (0, 2.5, 5, 10, 25, and 50 µg/mL) were prepared in ethanol to serve as standards for comparison. The standards, along with both single and sequential *S. glauca* leaf extracts of 1 mg/mL stock in ethanol, were incubated with 70 μL of 1:1 diluted (in water) Folin–Ciocalteu reagent and 60 μL of 0.4% sodium carbonate for 30 min at room temperature. Absorbance of developed color was measured at 760 nm using a microplate reader (PerkinElmer, Shelton, CT, USA). Ethanol acted as the blank, and the phenol content in each extract was calculated by reading the absorbance of unknown samples from a Gallic acid calibration curve. The results represent the mean of three independent batches of extracts with at least three replicate measurements in each batch.

#### 4.2.2. Quantification of Total Flavonoid Content (TFC)

The quantification of flavonoids in whole extracts was carried out in accordance with the procedures described by Mohammed et al., 2022 [67]. A series of Quercetin concentrations (0, 2.5, 5, 10, 25, 50, and 100 µg/mL) were utilized to establish a standard curve. The extract (60 μL) and the standards were incubated with 60 μL of 2% aluminum chloride for a duration of 60 min. The developed color was read at 420 nm, and the absorbance values of the extracts were compared against the standard curve to ascertain the TFC. The results represent the mean of three independent batches of extracts with at least three replicate measurements in each batch.

#### 4.2.3. RP-HPLC

The phenolic acid composition in the extracts was quantified using RP-HPLC (Prominence-i LC-2030C Shimadzu, Kyoto, Japan). In summary, a 1.0 mg/mL extract was prepared by dissolving it in a mobile phase composed of water, acetic acid, and methanol in an 80:5:15 ratio. This solution was then filtered and injected into a reverse phase C18 column (Shimpak, 4.6 × 250 mm), where it was eluted using an isocratic mobile phase at a flow rate of 1 mL/min. The identification and quantification of the phenolic acids were accomplished by comparing their relative retention times and areas to those of standard phenolic acids of 100.0 μg/mL. The standard phenolic acids included are Gallic acid (3,4,5-trihydroxy benzoic acid), Protocatechuic acid (3,4-Dihydroxy benzoic acid), p-Hydroxy benzoic acid (4-Hydroxy benzoic acid), Gentisic acid (2,5-Dihydroxy benzoic acid), Vanillic acid (4Hydroxy-3methoxy benzoic acid), Syringic acid (3,5-dimethoxy-4-Hydroxy benzoic acid), Veratric acid (3,4-Dimethoxy benzoic acid), Eudesmic acid (3,4,5-tri-methoxy benzoic acid), Benzoic acid, p-coumaric acid ((E)-3-(4-hydroxyphenyl)acrylic acid), Caffeic acid ((2E)-3-(3,4-dihydroxyphenyl)prop-2-enoic acid), Ferulic acid (3,4-Dihydroxycinnamic acid), Sinapic acid (3,5-Dimethoxy-4-hydroxycinnamic acid), Trans Cinnamic acid ((E)-3-phenylprop-2-enoic acid), 3,4-Dimethoxy cinnamic acid ((2E)-3-(3,4-dimethoxyphenyl)prop-2-enoic acid), and Chlorogenic acid (Cyclohexane-1-carboxylic acid) (Figure S1). An integrated UV detector was used to monitor the compounds at a wavelength of 254 nm [68,69].

### 4.3. Antioxidant Activity

#### 4.3.1. DPPH Free-Radical Scavenging Assay

The antioxidant properties of both single and sequential extracts from *S. glauca* leaves were evaluated using the DPPH free radical scavenging method. In summary, a 0.2 mM DPPH stock solution was prepared in 70% ethanol. Subsequently, 100 µL of this stock was combined with 100 µL of test solutions at various concentrations (6.25, 12.5, 25, 50, and 100 μg/mL), all dissolved in 70% ethanol. The mixtures were then incubated in the dark at room temperature for 30 min, after which the absorbance was measured at 517 nm using a microplate reader. The control used in this experiment was the 70% ethanol solution containing DPPH [70]. The radical scavenging activity was calculated as below.

Radical scavenging activity:

% inhibition = 1 − (A _sample_/_control_) × 100 (2)

where _control_ represents the absorbance of control and A_sample_ is the absorbance of the test.

#### 4.3.2. ABTS

The ABTS assay was performed following the methodology established in the previous study. The ABTS radical cation was generated by combining equal volumes of a 7 mM ABTS stock solution and a 2.45 mM potassium persulfate solution. This stock solution was then allowed to incubate in the dark at room temperature for a duration of 12–16 h to produce ABTS+•.

The generated radical cation was subsequently diluted with ethanol to achieve an absorbance of 0.700 at 734 nm. A mixture of 2 μL of *S. glauca* leaf extracts at a concentration of 1 mg/mL was combined with 200 μL of the ABTS+• solution and allowed to incubate in the dark for 10 min at room temperature. The absorbance was measured at 734 nm using a multi-plate reader. The 70% ethanol solution was taken as a control. The scavenging activity of ABTS radicals was calculated using standardized Equation (2) [71,72].

#### 4.3.3. Ferric Reducing Antioxidant Potential (FRAP)

The assay was performed in reference to the protocol of Shi et al., 2024 [73]. The working FRAP reagent was created by combining 300 mM acetate buffer (pH 3.6), 10 mM 2,4,6-tripyridyl-s-triazine (TPTZ) solution, and 20 mM FeCl_3_∙6H_2_O in a 10:1:1 ratio immediately prior to use. The 300 mM acetate buffer was formulated by dissolving 3.1 g of sodium acetate trihydrate in 16 mL of glacial acetic acid and diluting to a final volume of 1.0 L with distilled water. The TPTZ solution was prepared by dissolving 10 mM TPTZ in 40 mM HCl. For each well in a 96-well plate containing 10 µL of the test sample, 190 µL of the working FRAP reagent was added. The mixture was incubated for 30 min, after which the absorbance was measured at 593 nm.

### 4.4. Bioactivity Guided Fractionation

The cytotoxic potential of *S. glauca* leaf extracts was assessed using an MTT assay. CAL 27 cells were cultured in 96-well plates at a density of 8000 cells per well. Once the cells adhered, they were exposed to various concentrations (62.5, 125, 250, 500, and 1000 μg/mL) of individual and sequential extracts generated by using hexane, chloroform, ethyl acetate, ethanol, and water. After 24 h of incubation, 10 μL of 5 mg/mL MTT (HiMedia, Thane, India) was added and incubated for 3 h at 37 °C. Absorbance was measured at 570 nm using a multimode microplate reader (Multiskan GO, Thermo Scientific, Vantaa, Finland), and the percentage cytotoxicity, along with IC_50_, was calculated.

### 4.5. LC-MS Analysis

The chemical profiling of *S. glauca* leaf hexane extract (SGHE) was performed using a Thermo Scientific Vanquish UHPLC (Waltham, MA, USA) system coupled with a high-resolution mass spectrometer. Chromatographic separation was achieved on a Hypersil GOLD C18 reversed-phase column (100 mm × 2.1 mm, 1.9 µm particle size). The mobile phases consisted of (A) water containing 0.1% formic acid and (B) acetonitrile containing 0.1% formic acid. The flow rate was maintained at 0.3 mL/min with a total run time of 15 min. The gradient program was set as follows: 0–8 min, 5–95% B; 8–11 min, 95% B; 11–12 min, 95–5% B; and 12–15 min, 5% B for equilibration. The column temperature was maintained at 35 °C. The injection volume was 5.0 µL, and detection was carried out at 214 nm using a diode array detector.

### 4.6. Bioactivity Guided Fractionation and Enrichment

A 4.0 g portion of the crude hexane extract was subjected to silica-gel column chromatography [22]. Approximately 250 g of silica gel (packed volume ≈ 0.42–0.50 L) was used for packing the column. The loaded extract was eluted by using a mobile phase initially consisting of hexane:ethyl acetate (85:15). Fractions (48 tubes) were collected, and the solvent ratio was then adjusted to 75:25 until the 107th fraction, followed by a final elution at 50:50. Each fraction was collected in 15 mL portions. A total of 115 fractions were collected and analyzed by thin-layer chromatography (TLC). The fractions exhibiting identical banding patterns were pooled and concentrated using rotary evaporator (Buchi, Flawil, Switzerland) for further analysis, as shown in Figure S3.

The concentrated fractions were tested for bioactivity using CAL-27 cell line by an MTT assay, as explained above.

#### 4.6.1. Structural Characterization of Active Fraction

The fraction 44–55 exhibited greater cytotoxicity, and thus was characterized using LC-MS (SciEX, Triple TOF 6600+, Framingham, MA, USA), 1H and 13C NMR (JEOL 400 MHz, Tokyo, Japan).

#### 4.6.2. Bioactivity Examination of Active Fraction vs. Standard

The active fraction and standard compound (D-erythro-Sphinganine Sigma-Aldrich) were evaluated for cytotoxic activity using the CAL-27 cell line by SRB (Sulforhodamine B) assay. Experimentally, CAL-27 cells were cultured in 96-well plates at a density of 8000 cells per well. After 24 h, the exponentially growing cells were treated with different concentrations (62.5, 125, 250, 500, and 1000 μg/mL) of active fractions and standards (D-erythro-Sphinganine). After 24 h and 48 h of treatment, cells were fixed with 50 μL of cold 50% (*w*/*v*) TCA for 1 h at 4 °C. Later, the wells were washed with water (200 μL × 4 times) to remove TCA and serum proteins. The plates were air-dried and incubated with 100 μL 0.4% SRB (prepared in 1% acetic acid) for 30 min to stain the cellular proteins. The plates were washed with 1% acetic acid (200 μL) for 3–4 times to remove the unbound SRB. Tris base solution (10.0 mM, 100 μL/well) was used to solubilize the bound SRB, and the absorbance was measured in a multimode plate reader operating at 510 nm [74]. The percentage of cell viability was calculated using the equation shown below:

(3) %viability= 100− OD of control−OD of sampleOD of control ×100

### 4.7. Determination of the Impact of the Most Potent S. glauca Hexane Fraction on Cell Death Induction by Acridine Orange and Ethidium Bromide

The staining process for both live and dead cells utilized acridine orange and ethidium bromide [75]. A total of 0.5 × 10^6^ cells were seeded per well in a 6-well plate. Once the cells reached ~70% confluence, they were treated with three increased concentrations of the most potent fraction of hexane extract. The treatment concentrations were determined by the IC_50_ value. After 24 h and 48 h, the media was collected and the cells were trypsinized and pelleted. The pellet underwent a PBS wash, followed by the addition of 10 µL of each 100.0 µg/mL of acridine orange/ethidium bromide stain. The mixture was thoroughly combined, and 10 µL of the solution was placed on a slide. The cells were then observed under a fluorescence microscope using TRITC and FITC filters, and the images were merged to display live cells in green and dead cells in red.

### 4.8. Flow Cytometry Analysis

CAL-27 cells (0.5 × 10^6^ in 2 mL medium) were seeded in 6-well plates and incubated for 24 h at 37 °C in a CO_2_ incubator. Cells were then treated for 24 h with hexane extract (50, 100, and 200 µg/mL). Camptothecin (25 nM), 0.1% DMSO and untreated cells were served as the positive control, vehicle control, and negative control, respectively. After treatment, cells were collected, washed with PBS, and fixed by adding cold 70% ethanol dropwise while continuously mixing by gentle vortexing. The fixed cells were stored at 4 °C. For cell cycle analysis, fixed cells were washed twice with PBS, treated with RNase A (100 µg/mL), and stained with propidium iodide (PI; 50 µg/mL) for 30 min at room temperature in the dark. For apoptosis analysis, cells were resuspended in Annexin-V binding buffer and stained with Annexin V-FITC and PI for 15 min at room temperature. Samples were filtered through a 40 µm mesh and analyzed by flow cytometry (≥10,000 events/sample). Data acquisition, compensation, and analysis were performed using CellQuest Pro v6.0. Cell cycle distribution and apoptotic populations were quantified according to standard gating strategies [76].

### 4.9. Migration Assay

The migration assay was conducted to determine the ability of *S. glauca* hexane extract to inhibit the migration of CAL-27 cell line. Briefly, 0.5 × 10^6^ cells per well were seeded in 96-well plate. After 24 h, a scratch was made using 10 µL tip followed by PBS wash. Images were taken for 0 h, and the cells were treated with non-toxic concentration of *S. glauca* hexane extract (25, 50, and 75 µg/mL). Subsequently, the images were taken after 24 h and 48 h of treatment. The scratch area decreased due to cell migration was determined using Image-J software and the obtained value compared with respective baseline value to estimate the percentage inhibition of migration of cells. The higher the percentage inhibition of migrating cells, better the extract; hence, it can be considered as a potent anti-migratory agent/extract [76].

### 4.10. Statistical Analyses

Graph Pad Prism 8 was used to generate all figures. Data are presented as mean ± standard deviation (SD). Differences between the two groups were evaluated using Student’s *t*-test, while comparisons among three or more groups were assessed using one-way ANOVA. All the cell-based assays were carried out at least twice with at least two replicate measurements in each experiment.

## 5. Conclusions

*S. glauca* leaf extracts exhibited distinct phytochemical profiles and biological activities. Comprehensive phytochemical screening (TPC-, TFC-, and by RP-HPLC-based profiling), together with bioactivity assays, demonstrated that the ethyl acetate, ethanol, and water extracts exhibited stronger antioxidant activity (DPPH, FRAP, and ABTS) than the non-polar hexane extract. However, the non-polar hexane extract showed the highest anticancer potential against CAL-27, HeLa, and 4T1 cell lines. Bioactivity-guided fractionation of the hexane extract identified a major active fraction containing 93% D-erythro-Sphinganine; however, the crude hexane extract displayed superior overall activity, supporting the synergistic contribution of multiple constituents, likely triterpenoids and fatty acid derivatives. Mechanistic studies revealed that the hexane extract induced concentration-dependent disruptions in G_1_/S and G_2_/M transitions, increased both early and late apoptosis, and significantly inhibited cell migration, achieving ~60% wound closure inhibition at higher concentrations. Collectively, these findings indicate that the hexane extract of *S. glauca* leaves exerts multi-target anticancer effects and represents a promising candidate for further molecular and in vivo investigations.

## Figures and Tables

**Table 1:** IC-50 (μg/mL) of *S. glauca* leaf extracts on the oral, cervical, and breast cancer cell lines Cal-27, HeLa, and 4T1, respectively.

Cancer Cell Line Type	Cell Line	Sequential Extracts	IC_50_ Values of Sequential Extracts (µg/mL)		Single Extracts	IC_50_ Values of Single Extracts (µg/mL)	
			24 h	48 h		24 h	48 h
Oral cancer	CAL-27	SqHE	147	142	SiHE	256.1	190.8
		SqCE	603.3	131.4	SiCE	>1000	>1000
		SqEAE	724.4	230.3	SiEAE	308.3	189.0
		SqEtE	298.5	108.0	SiEtE	681.6	288.3
		MSqWE	>1000	490.4	MSiWE	>1000	760.4
Cervical Cancer	HeLa	SqHE	>1000	459	SiHE	>1000	299
		SqCE	>1000	>1000	SiCE	>1000	>1000
		SqEAE	>1000	742	SiEAE	>1000	>1000
		SqEtE	>1000	>1000	SiEtE	>1000	>1000
		MSqWE	>1000	>1000	MSiWE	930.9	265
Breast Cancer	4T1	SqHE	>1000	258	SiHE	650	230
		SqCE	>1000	>1000	SiCE	>1000	>1000
		SqEAE	>1000	302	SiEAE	>1000	523.8
		SqEtE	>1000	542	SiEtE	502	132.7
		MSqWE	>1000	>1000	MSiWE	>1000	>1000

## Data Availability

The original contributions presented in this study are included in the article/Supplementary Material. Further inquiries can be directed to the corresponding authors.

## Supplementary Materials

The following supporting information can be downloaded at https://www.mdpi.com/article/10.3390/molecules31030497/s1. Figure S1: RP-HPLC standards - Benzoic acids and cinnamic acids; Figure S2: RP-HPLC elution profiles of single and sequential extracts of *S.glauca* leaves; Figure S3: Cytotoxic effect of single and sequential extracts on Beas-2B cells; Figure S4: Thin layer chromatography; Figure S5: Cytotoxicity of the fractions of hexane extract of *S. glauca* leaves; Figure S6: Cytotoxicity of the active fractions (44–55) and standard compound (D-erythro-sphinganine); Table S1: Quantified compounds in the Hexane Extract of *S. glauca* by Using LC-HRMS title.

## Abbreviations

The following abbreviations are used in this manuscript:

OSCC	Oral Squamous Cell Carcinoma
SiHE	Single hexane extract
SqHE	Sequential hexane extract
SGHE	*Simarouba glauca* hexane extract
SiCE	Single chloroform extract
SqCE	Sequential chloroform extract
SiEAE	Single ethyl acetate extract
SqEAE	Sequential ethyl acetate extract
SiEtE	Single ethanol extract
SqEtE	Sequential ethanol extract
MSiWE	Macerated single water extract
MSqWE	Macerated sequential water extract
TPC	Total Phenolic Content
TFC	Total Flavonoid Content
FRAP	Ferric ion Reduction Antioxidant Power
DPPH	2,2-diphenyl-1-picrylhydrazyl
ABTS	2,2′-azinobis-(3-ethylbenzothiazoline-6-sulfonic acid)
MTT	3-(4,5-dimethylthiazol-2-yl)-2,5-diphenyltetrazolium bromide
RT	Retention time
ROS	Reactive Oxygen Species
ANT	Adenine nucleotide translocase
