# Supplementary material for: Bioactivity-Guided Fractionation, Characterization, and Mechanistic Insights of Anticancer Agents from Simarouba glauca DC. Leaves
Source: Molecules. 2026 Jan 31;31(3):497. doi: 10.3390/molecules31030497 (PMC12900014; doi:10.3390/molecules31030497)
Supplement: Supplementary file 1 [file molecules-31-00497-s001.zip › molecules-4096821-supplementary.pdf]

Supplementary Results

List of Contents

Figure S1: RP-HPLC standards - Benzoic acids and cinnamic acids 2-3

Figure S2: RP-HPLC elution profiles of single and sequential extracts of *S.glauca* leaves 4-5

Figure S3: Cytotoxic effect of single and sequential extracts on Beas-2B cells……………….6-7

Figure S4: Thin layer chromatography…………………………………………………………8

Figure S5: Cytotoxicity of the fractions of hexane extract of *S. glauca* leaves…………………9

Figure S6: Cytotoxicity of the active fractions (44-55) and standard compound (D-erythro-sphinganine) ……………………………………………………………………………………….9

Table S1: Quantified compounds in the Hexane Extract of *S. glauca* by Using LC-HRMS…..10

Supplementary Figure S1

A.

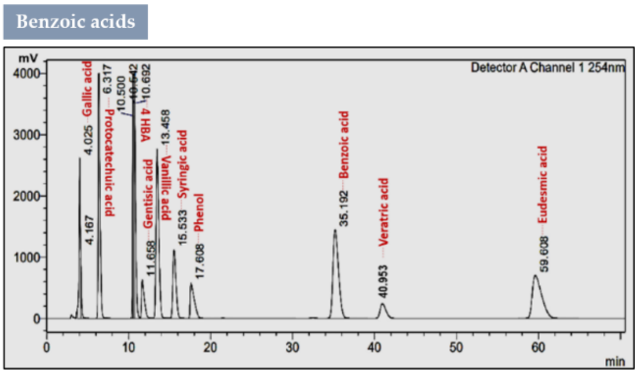

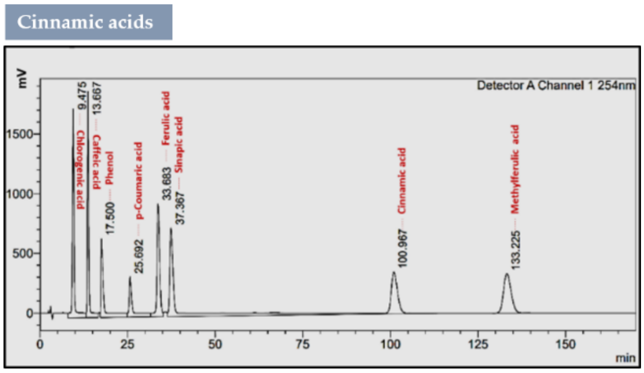

B.

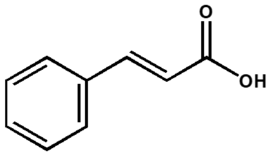

**Cinnamic acid**
**(2E)-3-phenylprop-2-enoic acid**
**$C_9H_8O_2$**
**148.16 g/mol.**

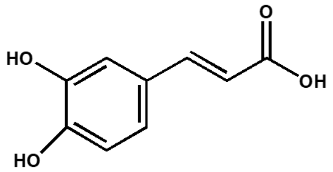

**Caffeic acid**
**(E)-3-(3,4-dihydrohenyl)acrylic acid**
**$C_9H_8O_4$**
**180.16 g/mol**

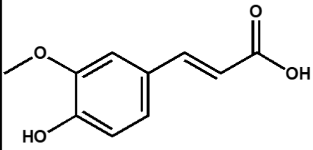

**Ferulic acid**
**(E)-3-(4-hydroxy-3-methoxyphenyl)acrylic acid**
**$C_{10}H_{10}O_4$**
**194.18 g/mol**

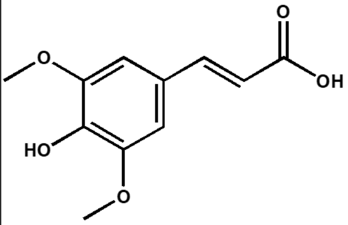

**Sinapic acid**
**(E)-3-(4-hydroxy-3,5-dimethoxyphenyl)acrylic acid**
**$C_{11}H_{12}O_5$**
**224.21 g/mol**

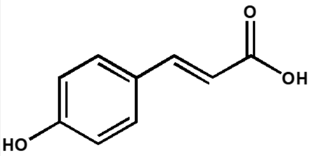

**P-coumaric acid**
**(E)-3-(4-hydroxyphenyl)acrylic acid**
**$C_9H_8O_3$**
**164.16 g/mol**

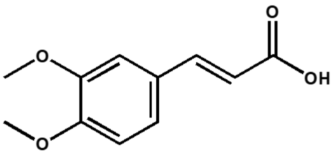

**3, 4- dimethoxy cinnamic acid**
**(E)-3-(3,4-dimethoxyphenyl)acrylic acid**
**$C_{11}H_{12}O_4$**
**208.21 g/mol**

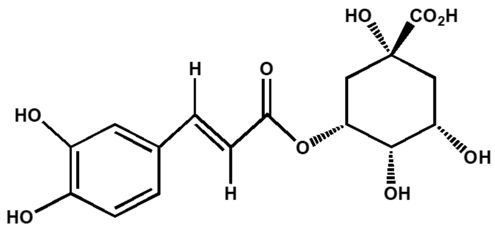

**Chlorogenic acid**
**(1S,3R,4R,5R)-3-[(E)-3-(3,4-dihydroxyphenyl)prop-2-enoyl]oxy-1,4,5-trihydroxycyclohexane-1-carboxylic acid**
**$C_{16}H_{18}O_9$**
**354.31 g/mol**

C.

Structure	Name	Systematic name	Formula	Molar mass
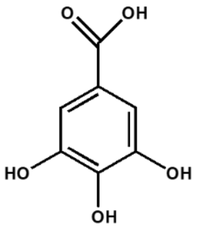	Gallic acid	3,4,5-trihydroxybezoic acid	$C_7H_6O_5$	170.12 g/mol
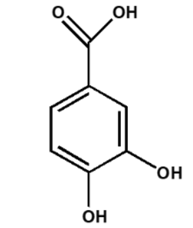	Protocatechuic acid	3,4-dihydroxybenzoic acid	$C_7H_6O_4$	154.12 g/mol
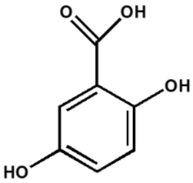	Gentisic acid	(2,5-dihydroxybenzoic acid)	$C_7H_6O_4$	154.12 g/mol
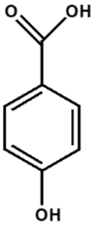	p-hydroxybenzoic acid	4-hydroxybenzoic acid	$C_7H_6O_3$	138.12 g/mol
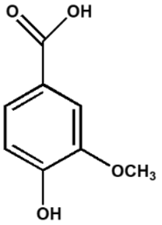	Vanillic acid	4-hydroxy-3-methoxybenzoic acid	$C_8H_8O_4$	168.15 g/mol
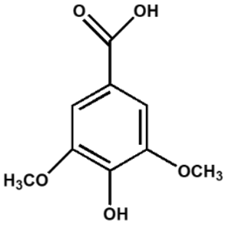	Syringic acid	4-hydroxy-3,5-dimethoxybenzoic acid	$C_9H_{10}O_5$	198.17 g/mol
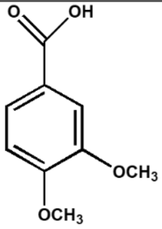	Veratric acid	3,4-dimethoxybenzoic acid	$C_9H_{10}O_4$	182.17 g/mol
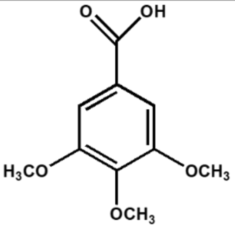	Eudesmic acid	3,4,5-trimethoxy-benzoic acid	$C_{10}H_{12}O_5$	212.20 g/mol
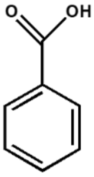	Benzoic acid	Benzoic acid	$C_7H_6O_2$	122.12 g/mol

Figure S1. A. RP-HPLC elution profiles of Hydroxy and methoxy benzoic and cinnamic acid standards, B. Cinnamic acid standards, and C. Benzoic acid standards

Supplementary Figure S2

A.

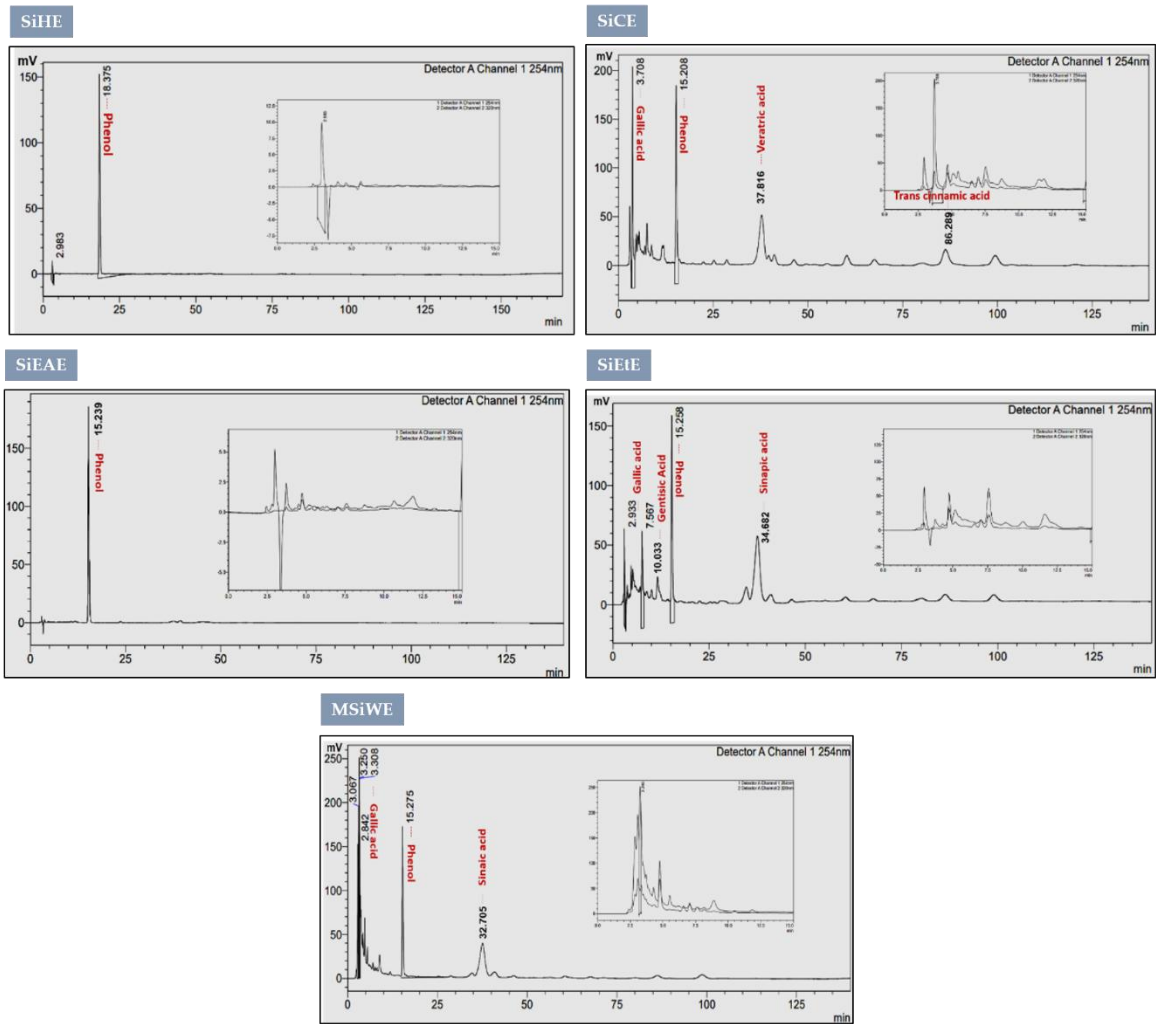

B.

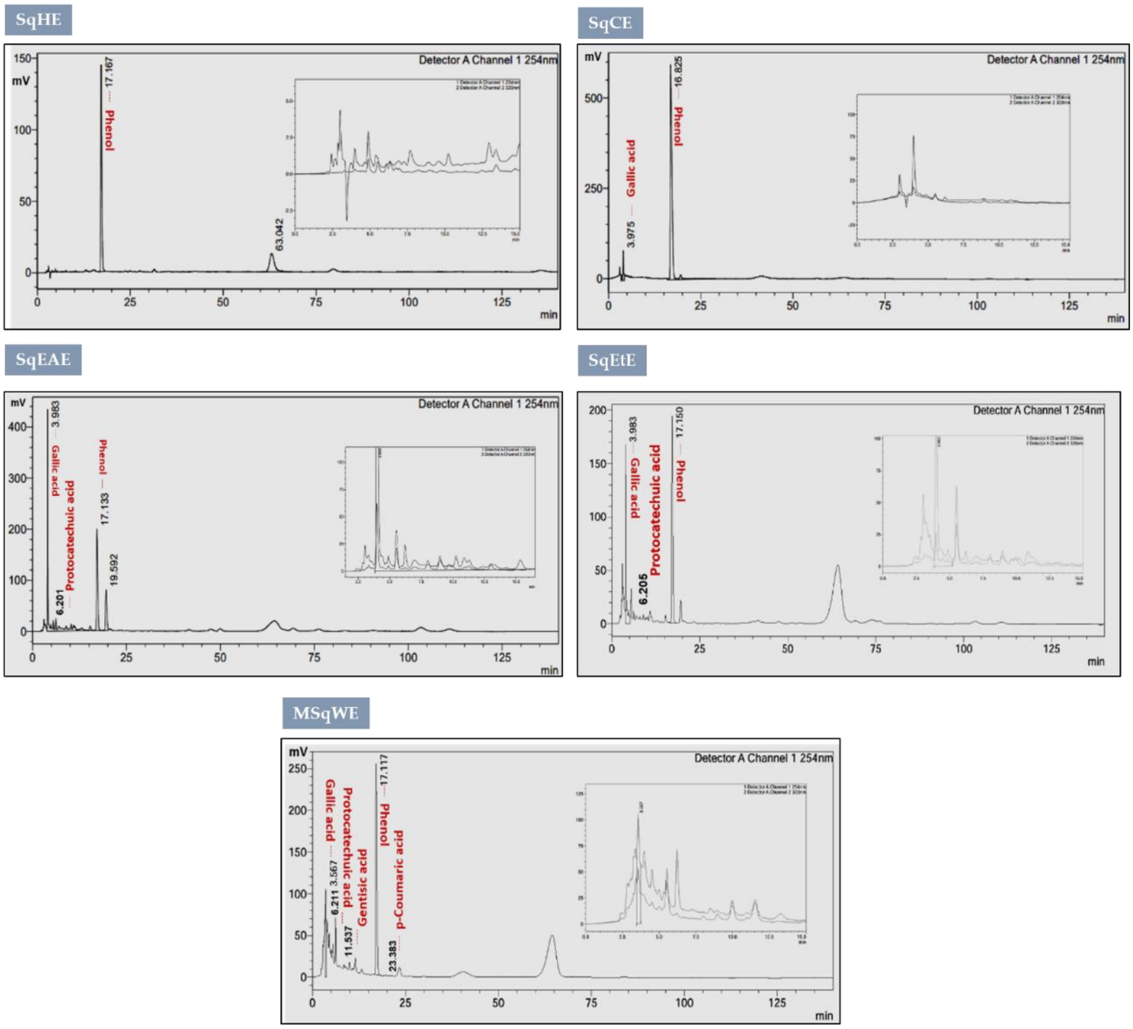

Figure S2. RP-HPLC elution profiles of single and sequential extracts of S.glauca leaves: RP-HPLC of A. SIHE: Single hexane extract, SICE: Single chloroform extract, SIEAE: Single ethyl acetate extracts, SIETE: Single ethanol extracts, MSIWE: Macerated Single water extract, and B. Sequential extracts (SQHE: Sequential hexane extract, SQCE: Sequential chloroform extract, SQEAE: Sequential ethyl acetate extracts, SQETE: Sequential ethanol extracts, MSIWE: Macerated Sequential water extract).

Supplementary Figure S3

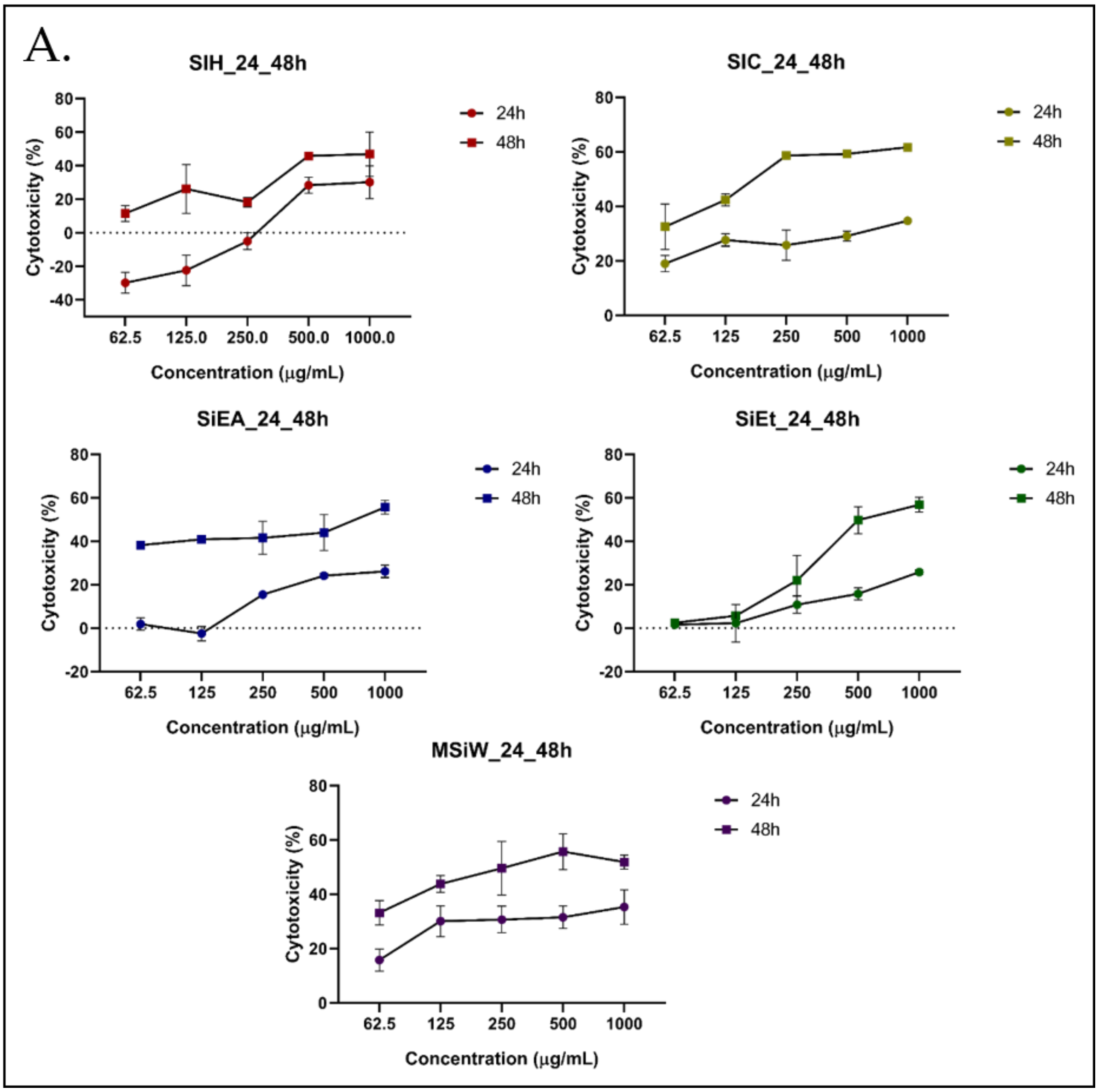

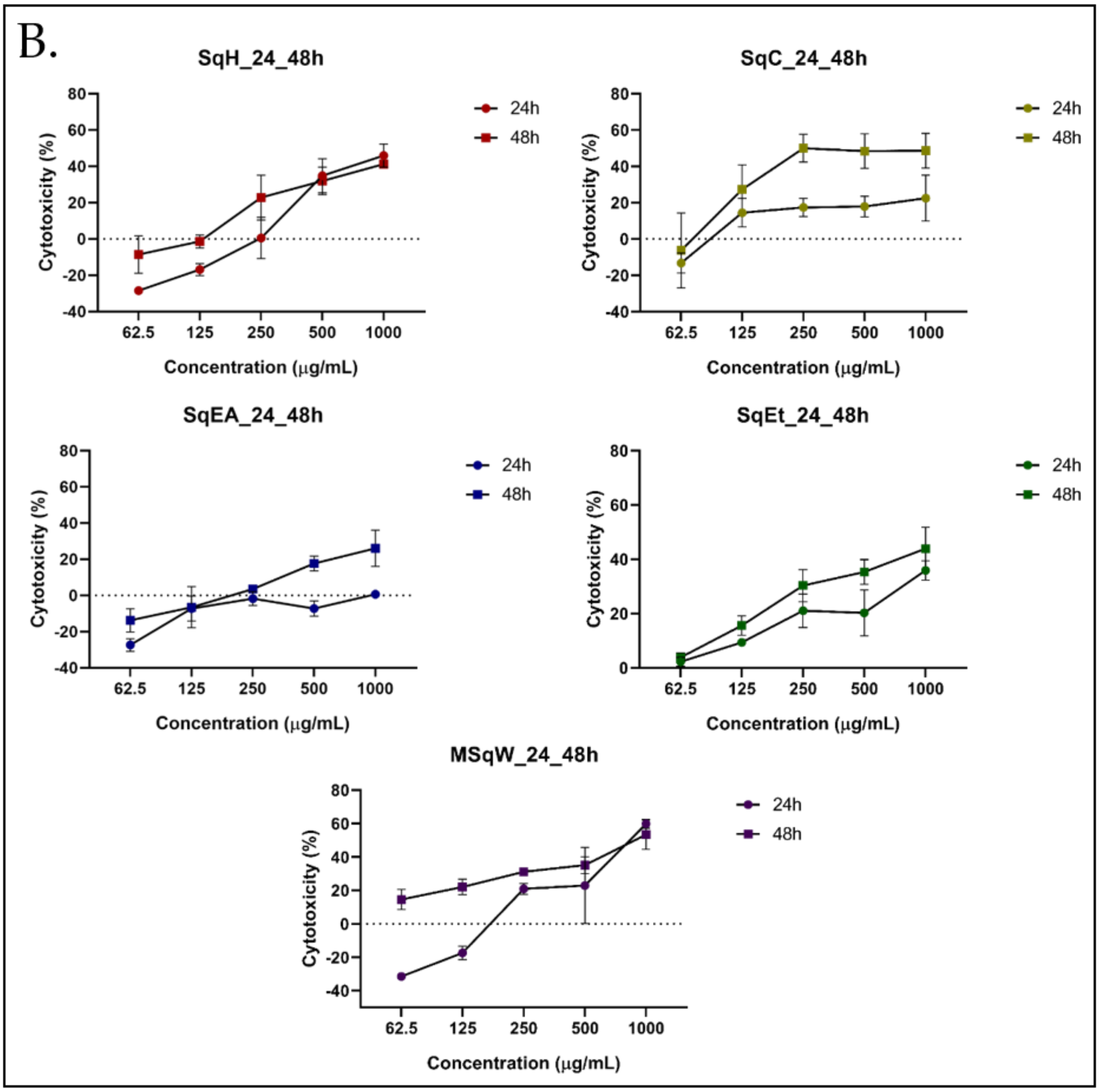

Figure S3. Cytotoxic effect of single and sequential extracts on Beas-2B cells: A. Single extracts, and B. Sequential extracts from *S. glauca*

Supplementary Figure S4

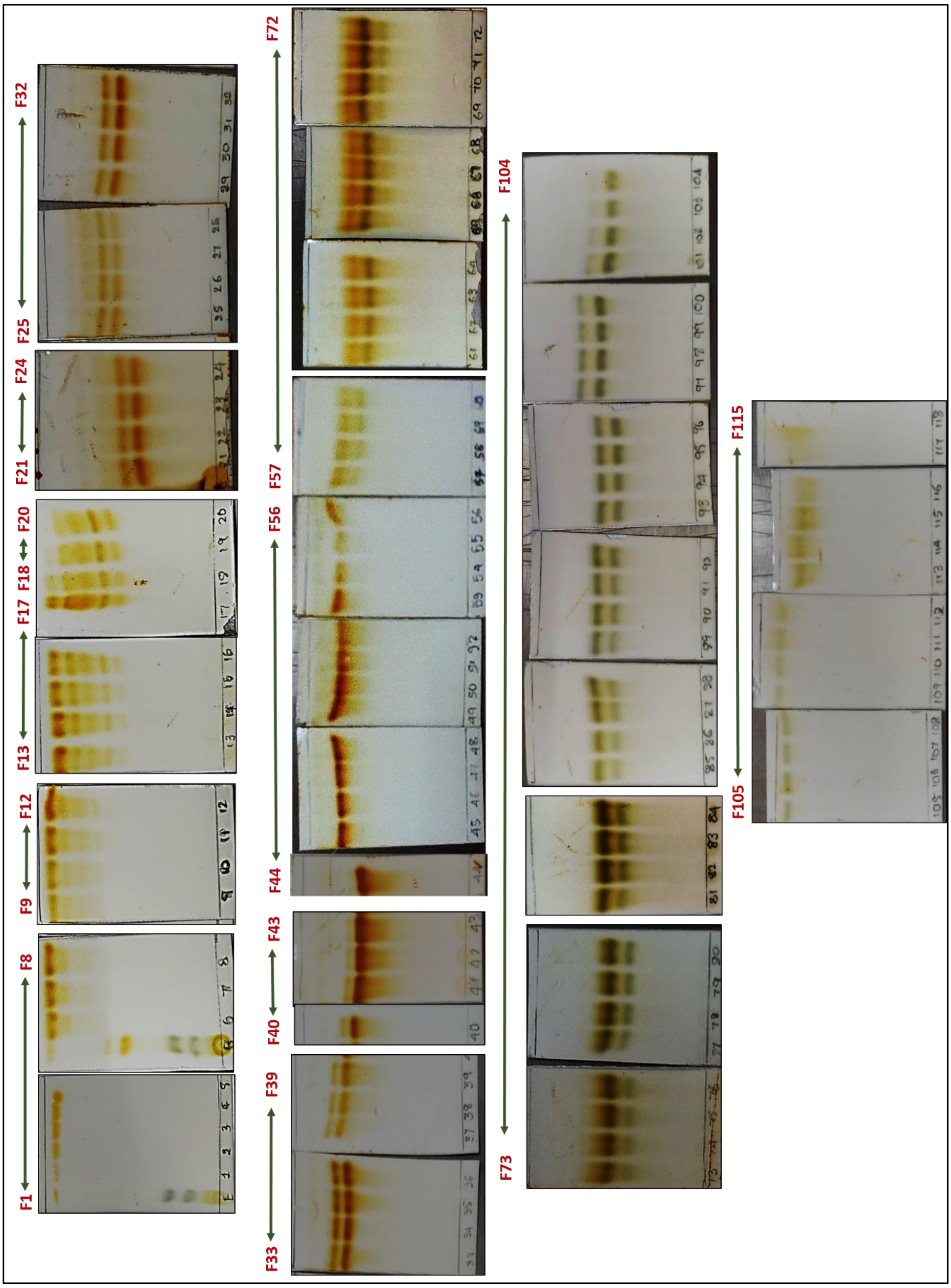

Figure S4. Thin layer chromatography: TLC of the collected fractions from 1- 115 using hexane and ethyl acetate as mobile phase in different ratios.

Supplementary Figure S5

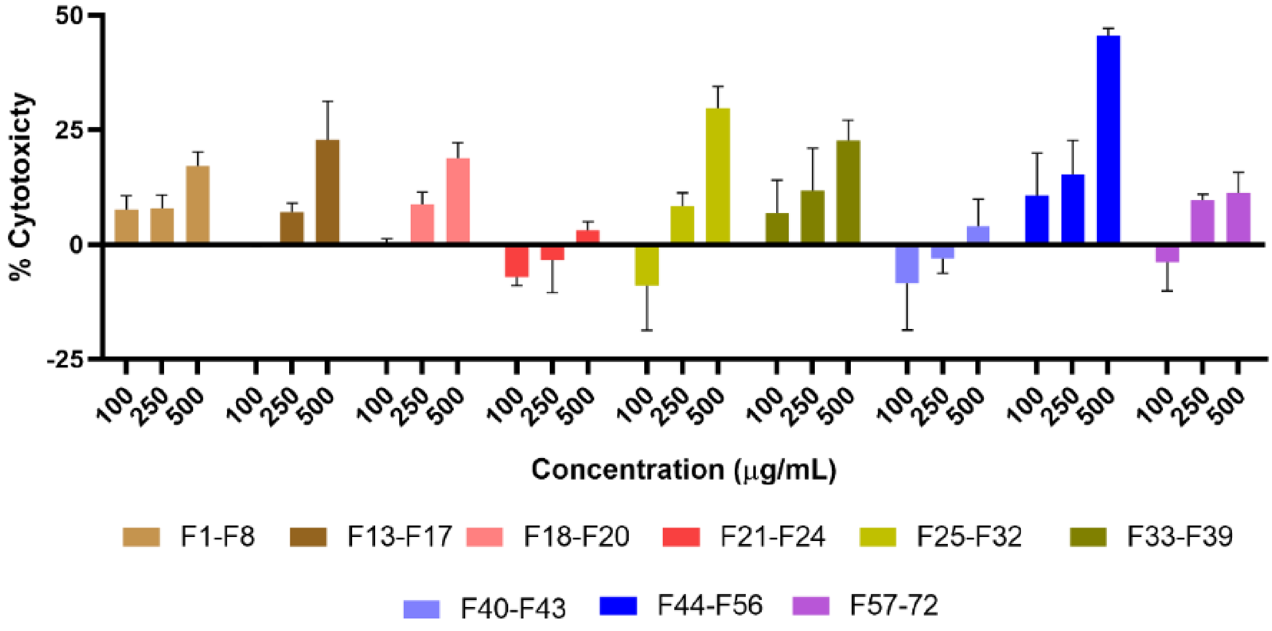

Figure S5. Cytotoxicity of the fractions of hexane extract of *S. glauca* leaves: Cytotoxic effects of fractions of hexane extract on CAL-27, Oral Squamous Cell Carcinoma, where cells were treated with different concentrations of extracts for 48 h, were measured using MTT assay at 570 nm

Supplementary Figure 6

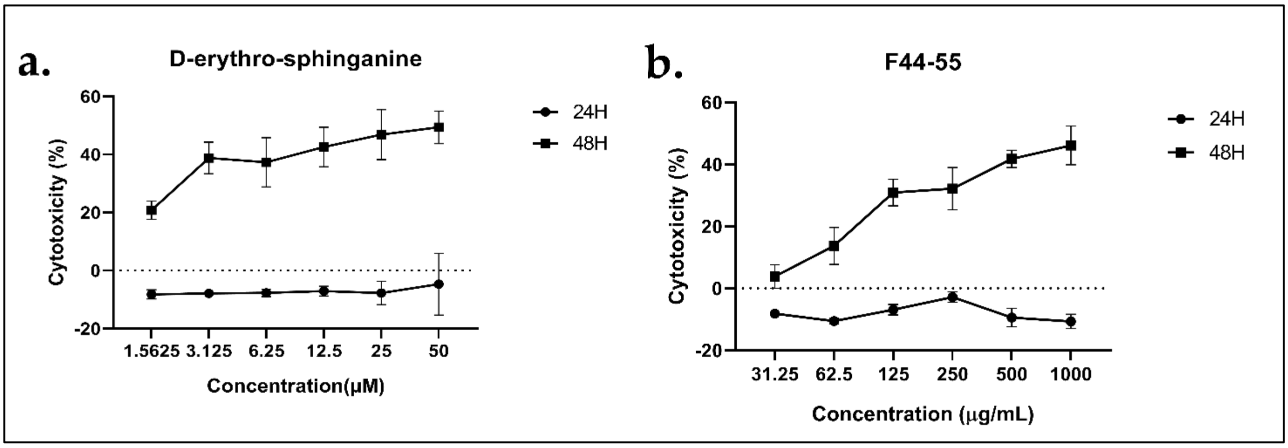

Figure S6. Cytotoxicity of the active fractions (44-55) and standard compound (D-erythro-sphinganine). Cytotoxic effects of a. D-erythro-sphinganine and b. F44-55, potent fraction on CAL-27 cell line, where cells were treated with different concentrations of extracts for 24 and 48 h, were measured using SRB assay at 510 nm

Supplementary Table S1

Table S1: Quantified compounds in the Hexane Extract of *S. glauca* by Using LC-HRMS

Sl no.	Compounds	RT (minutes)	Area	MW (Daltons)	Formula	Class / Type
1	(10E,12Z)-9-Hydroperoxy-10,12-octadecadienoic acid	8.820556	30331828	312.2298	$C_{18}H_{32}O_4$	Oxylipin / hydroperoxy fatty acid
2	Embelin	8.671389	25872550	294.1828	$C_{17}H_{26}O_4$	Quinone / Secondary metabolite
3	Ricinoleic Acid	10.84061	7201915	298.2501	$C_{18}H_{34}O_3$	Hydroxy fatty acid
4	Propapyriogenin A2	10.011	13171148	484.3189	$C_{30}H_{44}O_5$	Triterpenoid / saponin derivative
5	Linolenelaidic acid	10.84617	8396700	278.2242	$C_{18}H_{30}O_2$	Unsaturated fatty acid
6	Juniperic acid	10.09506	4234115	272.2349	$C_{16}H_{32}O_3$	Long-chain fatty acid
7	AGARICIN	9.635333	4870169	416.277	$C_{22}H_{40}O_7$	Triterpenoid / natural product
8	Palmitic Acid	12.17333	3440554	256.2399	$C_{16}H_{32}O_2$	Saturated fatty acid
